# Emerging Adaptive Strategies Under Temperature Fluctuations in a Laboratory Evolution Experiment of *Escherichia Coli*

**DOI:** 10.3389/fmicb.2021.724982

**Published:** 2021-10-22

**Authors:** Maryl Lambros, Ximo Pechuan-Jorge, Daniel Biro, Kenny Ye, Aviv Bergman

**Affiliations:** ^1^Department of Systems and Computational Biology, Albert Einstein College of Medicine, Bronx, NY, United States; ^2^Department of Epidemiology and Population Health, Albert Einstein College of Medicine, Bronx, NY, United States; ^3^Dominick P. Purpura Department of Neuroscience, Albert Einstein College of Medicine, Bronx, NY, United States; ^4^Department of Pathology, Albert Einstein College of Medicine, Bronx, NY, United States; ^5^Santa Fe Institute, Santa Fe, NM, United States

**Keywords:** experimental evolution, fluctuating environmental temperatures, adaptation strategies, *Escherichia coli*, laboratory evolution, evolutionary contingency

## Abstract

Generalists and specialists are types of strategies individuals can employ that can evolve in fluctuating environments depending on the extremity and periodicity of the fluctuation. To evaluate whether the evolution of specialists or generalists occurs under environmental fluctuation regimes with different levels of periodicity, 24 populations of *Escherichia coli* underwent laboratory evolution with temperatures alternating between 15 and 43°C in three fluctuation regimes: two periodic regimes dependent on culture's cell density and one random (non-periodic) regime with no such dependency, serving as a control. To investigate contingencies on the genetic background, we seeded our experiment with two different strains. After the experiment, growth rate measurements at the two temperatures showed that the evolution of specialists was favored in the random regime, while generalists were favored in the periodic regimes. Whole genome sequencing demonstrated that several gene mutations were selected in parallel in the evolving populations with some dependency on the starting genetic background. Given the genes mutated, we hypothesized that the driving force behind the observed adaptations is the restoration of the internal physiology of the starting strains' unstressed states at 37°C, which may be a means of improving fitness in the new environments. Phenotypic array measurements supported our hypothesis by demonstrating a tendency of the phenotypic response of the evolved strains to move closer to the starting strains' response at the optimum of 37°C, especially for strains classified as generalists.

## 1. Introduction

Fluctuating environmental conditions are hypothesized to play a major role in the evolution of complex adaptive traits (Pál and Papp, 2017). The spatially heterogeneous and dynamic nature of the environment, encompassing both biotic and abiotic factors, such as temperature, is thought to be one of the evolutionary drivers behind the high diversity of species that have populated our biosphere over time (Fuentes and Ferrada, 2017). Environmental complexity, besides the connatural potential of living systems (Ruiz-Mirazo et al., 2008), is thought to help explain the seemingly open ended nature of the evolutionary process. The characteristics of environmental fluctuations, like random vs. periodic, play a fundamental role in the specific ways biological organisms adapt to different selective pressures that vary dynamically or spatially (Kussell and Leibler, 2005; Botero et al., 2015). Adaptations to environmental complexity take the form of a variety of structures, life-cycles, behaviors, and ultimately molecular mechanisms that contribute to the diversity of our biosphere. There are two main strategies when facing environmental complexity: (1) adapting to one or few environments, namely becoming specialists, or (2) adapting to a larger subset of the possible environments, namely becoming generalists. The granularity and the periodicity of environmental change can determine the evolution of generalists or specialists (Futuyma and Moreno, 1988; Brown and Pavlovic, 1992). For temporal environmental complexity, like the alternation of seasons, the periodicity of the fluctuation pattern determines the adaptive strategy that will primarily evolve, such that periodic fluctuations favors the evolution of generalists. However, the role of the underlying genetics in determining this is not well-understood.

Although both ecological and paleontological data can be used to assess these hypotheses and predict evolutionary strategies, adaptive laboratory evolution experiments provide a controlled setting where tenets about the evolutionary process can be tested on model organisms whose biology is known in great detail (Elena and Lenski, 2003; Herron and Doebeli, 2013). Even though the results derived from these laboratory experiments might be hard to extend to the more complex natural environments, they provide us with observations that can potentially be understood at the molecular level and give new insights that can be used to construct better theoretical frameworks. One of the most commonly used laboratory evolution organisms is *Escherichia coli*, and many questions regarding the dynamics of adaptation to different environmental stresses have been formulated with the set-up that Lenski and co-workers established as the pioneers of modern era experimental evolution (Lenski et al., 1991; Barrick et al., 2009). This set-up has been extensively used together with genome sequencing to explore a manifold of adaptive scenarios involving different degrees of complex environmental conditions to which the ancestral strain was not adapted (Satterwhite and Cooper, 2015).

In this work, we made use of batch culture laboratory evolution of *E. coli* under fluctuating temperatures as a simplified model of evolution under dynamic environmental complexity to survey if periodicity vs. not of environmental fluctuations affects the types of adaptive strategies that evolve in replicate populations. Having two environmental fluctuation regimes with different rates of periodicity and a third control fluctuation regime that is random with no periodicity, along with several replicates per fluctuation regime, allowed us to identify evolutionary parallelism and assess how periodicity affects the evolved responses. The model organism, *E. coli*, is well-adapted to grow at 37°C and has shown its potential for adapting to a variety of environmental challenges. Temperature was chosen as the environmental variable to fluctuate because of its easy manipulation, and the extensive availability of literature on laboratory evolution experiments with *E. coli* at constant temperatures that are different from the optimum at 37°C (Riehle et al., 2001; Kishimoto et al., 2010; Rudolph et al., 2010; Tenaillon et al., 2012; Sandberg et al., 2014) and some literature considering temperature fluctuations (Bennett et al., 1992; Ketola et al., 2013; Saarinen et al., 2018). In our experiment, the culture temperature was alternated between 15 and 43°C as they are extreme yet survivable values of the *E. coli* temperature niche. Given the different physiological states required to address extreme low and high temperatures, these two temperatures can be considered effectively separate environments in the same way different carbon sources would be.

**Figure 1:**
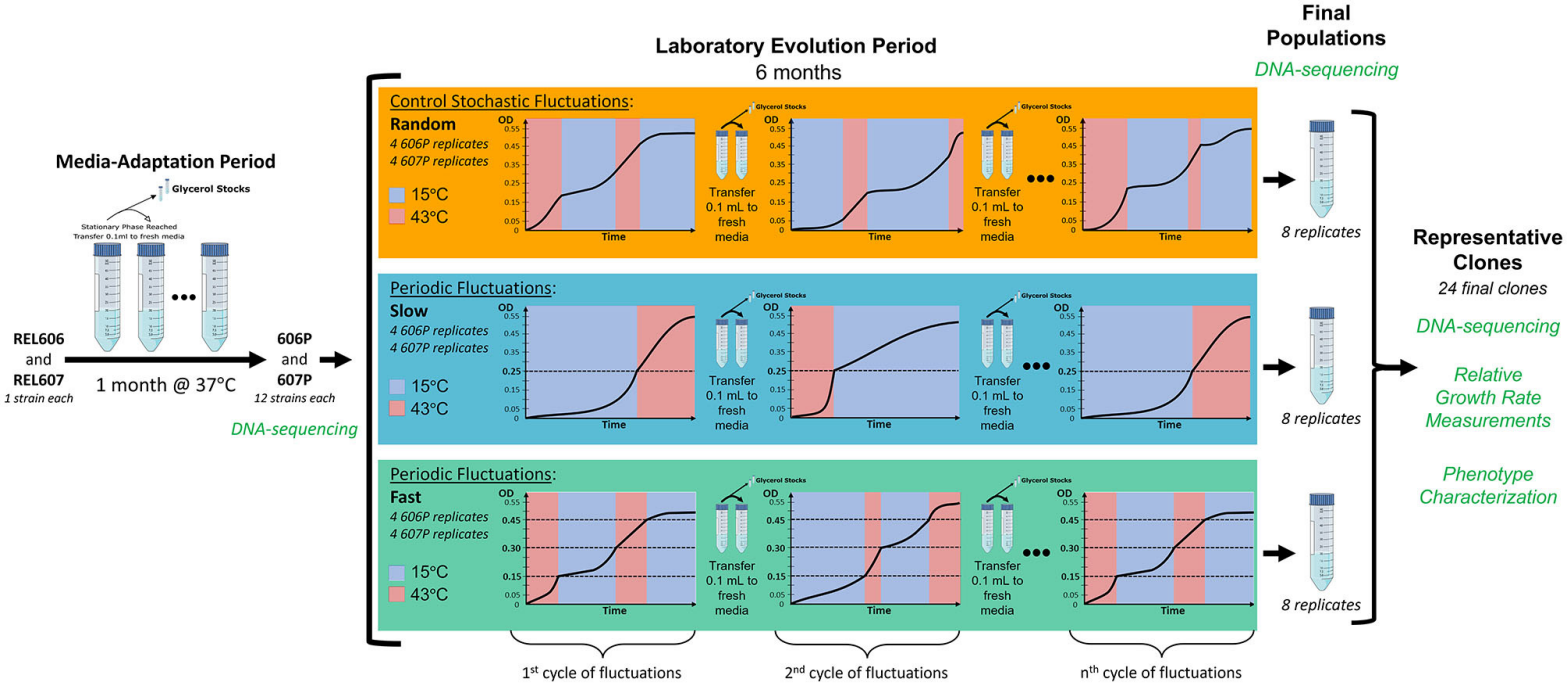
Schematic of our laboratory evolution experiment in fluctuating environments. Dashed lines represent the optical densities when temperatures where switched in the periodic treatments. To ensure similar times are spent in each growth curve phase for each of the two temperatures we are fluctuating between, the fluctuation regimes start a new cycle at the temperatures they were transferred from in the previous cycle. In other words, we alter which temperature each fluctuation cycle begins with.

In order to evaluate the effect of the scale and periodicity of the environmental changes on the evolution of our populations, we employ three environmental regimes that undergo cycles of fluctuations between 15 and 43°C. Two of these regimes have cycles that fluctuate in a periodic fashion as a function of the optical density (OD) of the culture with two different generation time scales, one fast and one slow. The third regime serves as a control and has cycles that randomly fluctuate between the temperatures with respect to time and OD, and so is not a periodic regime (for a detailed schematics of the evolution experiment see Figure 1). Right before stationary phase is reached at the end of each cycle of fluctuation, transfer to fresh media is done so that selection is based on the temperature fluctuation regime. The transition time to shift between the two temperatures is 35 min or less (see Supplementary Figure 1). Since the strains spend less than one generation in the temperature shift, the temperature transition should not be a driving force of the evolution. Additionally, we alternate the starting temperature of each cycle of fluctuation.

The choice to dynamically switch environmental treatments is predicated on the different adaptive responses that are conjectured to dominate in these different environments. The two periodic regimes (termed slow and fast), in principle, should favor the evolution of a generalist strategy because the variants that adapt to both environments will be favored since these fluctuation regimes are periodic and the population must grow a certain amount in each temperature. Here, a strain is classified as a “generalist” if the relative growth rate statistically significantly increased at both 15 and 43°C (meaning at least a 15 or 30% increase in relative growth rate in each temperature, respectively), classified as “specialist” if relative growth rate increased in only one temperature, and classified as “not-significant” if relative growth did not statistically significantly increase in either temperature (suggesting not adaptative at either temperature). Note, our definition of a generalist is the least biased definition such that it avoids applying an arbitrary threshold on the amount of increase in growth rate at each temperature. Additionally, since the level of adaptation to the environmental regimes can be reflected in the amount of varation of the evolved strains' relative growth rates at each of the two experimental temperatures, “generalists” should have increased variation in both temperatures and “specialists” should have increased variation in only one temperature that it is a specialist in, which we observe in our evolved strains.

The two periodic treatments differ in the optical density that triggers the temperature change and thus in the amount of time that the population experiences at a given temperature segment for one cycle of the fluctuation regime, allowing us to assess the effects of the length of time spent in each temperature condition on the types of adaptive responses evolved. The populations must grow well in both temperatures to make it to the OD value that triggers a change in temperature. Moreover, the periodic nature of these two fluctuating regimes can be potentially internalized by the organism. The stochastic treatment serves as a control where the number of generations that experience a certain environment does not depend on the population density. Thus, populations in the stochastic environment are not required to grow a certain amount to trigger a temperature change, but merely survive a random amount of time in each temperature. It could be argued that a constant temperature experiment at each of the two temperatures of the fluctuation is required. While this would provide an insight into the adaptive response to each of these environments separately, it ignores the constraint and potential trade-off due to the experience of the other environment and can lead to a level of specialization not observed in a complex environment with fluctuations. Furthermore, there is no choice for the organism to adapt under a constant temperature so it is forced to become a specialist in the absence of environmental changes.

Additionally, to investigate the dependency of the evolutionary outcome on the genetic background, the starting populations were derived from REL606 and REL607 strains adapted to the media at their optimal temperature of 37°C during the media-adaptation period of our experiment (see Figure 1). This media-adaptation period also served as a means of removing the adaptive impact of our particular culture conditions in the whole evolutionary outcome and was confirmed by the early appearance of mutations previously associated with adaptation to glucose minimal media.

The outcome of the laboratory evolution period in terms of adaptive strategies was evaluated by measuring the relative growth rate of the representative clones from the final evolved strains with respect to their starting population, demonstrating the preferential evolution of generalists in the periodic regimes. We found that the genetic background seemingly constrains the adaptive strategies that can evolve. Additionally, whole genome sequencing of the final populations and representative clones pointed toward genetic adaptations causing tuning of the proteome and the proteostatic machinery to the new environmental conditions, with amplifications impacting heat-shock machinery, translation, and transcription. Thus, we further hypothesized that the accumulated mutations would result in stabilizing the evolved strain's proteome in the stressful environmental conditions and thus promote the restoration of the internal cell physiology to that of the starting strains in the optimum environment of 37°C. In other words, the mutations that accumulate during adaptation may act to restore the evolved strains' phenotypes from a stressed to a pre-stressed condition, as observed by Hug et al. (2016) under adaptation to constant 42.2°C temperature. So, the genetic and phenotypic adaptation occurring in the longer term could work to shift the initial stressed and sub-optimal cellular physiology to the pre-stressed physiological state with higher growth resembling that of the ancestor's state in the optimum condition. This restoration of the phenotype to a pre-stressed state was found to occur when *E. coli*. strains were adapted to a constant hot temperature (Hug et al., 2016). We evaluated this hypothesis by observing the evolved changes in the internal cell state, as given by a 96 well phenotypic assay with many different conditions, and confirming that after laboratory evolution under fluctuating environments, most changes approximate the evolved strain's state to that of the starting population's internal cell state at the optimal condition.

## 2. Results

### 2.1. Media-Adaptation to Culture Media Leads to Mutations in Metabolic Related Genes

We hypothesized that during the early stages of the laboratory evolution experiment some highly advantageous mutations would fix as a consequence of providing a fitness increase related to growth in glucose minimal media. In order to reduce the impact of such mutations during the laboratory evolution experiment, we performed a media-adaptation step for a month to the glucose minimal media with the two *E. coli* strains obtained from Lenski, REL606, and REL607, at 37°C. REL606 and REL607 only differ by two neutral mutations, one in the *araA* gene that is used to distinguish between the two strains to ensure no cross-contamination occurs during the laboratory evolution experiment, and one in the *recD* gene that is also seen in Tenaillon et al. (2016) that arose inadvertently. The resulting media-adapted populations, which we call 606P and 607P, are used as the starting points for our laboratory evolution experiment under three different fluctuating temperature regimes between 15 and 43°C (see Figure 1). Any genomic changes in the populations are determined by whole genome sequencing at a high depth of at least 6,000 x. The fitness impact of the adaptation period is evaluated by measuring the relative change in growth rates of the resulting media-adapted starting populations, 606P and 607P, to the original strains, REL606 and REL607, at the three temperatures used in the experiment. The resulting media-adapted strains provide a baseline for the easily acquired adaptive mutations to the culture media. It is important to note that we started with the original Lenski strains and performed a media-adaptation period, instead of starting with one of the descendants of the LTEE strains that had evolved for almost 70,000 generations, because these highly evolved strains were grown in media with such a minimal amount of glucose that growth at 15°C would be too slow to be practical for our laboratory evolution experiment.

The whole genome sequencing data shows that most novel mutations reached fixation or high frequencies during media adaptation (see Supplementary Table 1). Predominantly, the mutations impact genomic regions related to metabolism, and most of the genes impacted have been documented during evolution in similar experiments (Phaneuf et al., 2019). Additionally, the media-adapted strains exhibit an increased growth rate compared to the original Lenski strains at 37°C (see Supplementary Figure 2). The early appearance and fixation of these mutations together with the relative growth rate increase observed after media adaptation suggest that the mutations are adaptive to the glucose minimal media at the optimal temperature of 37°C. However, we cannot rule out that some mutations may be neutral hitchhiking mutations.

Strikingly though, the two media-adapted populations have very different evolutionary trajectories with respect to the acquired mutations, which is also reflected in the growth rate gains at the unstressed temperature (37°C) and the temperatures that will be fluctuating between during the laboratory evolution experiment period (15 and 43°C). Specifically, the impact on fitness during media adaptation is represented by an increase in the growth rates of both the media-adapted populations at 37 and 43°C relative to the original REL606 and REL607 strain each was derived from. However, only the 606P population has an increase in growth rate at 15°C (see Supplementary Figure 2). This growth rate increase in the two temperatures the populations were not exposed to during media adaptation, 15 and 43°C, suggests that adaptation to the media at the optimal temperature of 37°C can also lead to changes in the response to other temperatures. This observation helps justify the media-adaption period prior to our laboratory evolution experiment in which the strains will be exposed to 15 and 43°C. Overall, the 606P population has a significantly higher growth rate than 607P at both 15 and 37°C. In accordance with the growth rates, 606P has a greater number of potentially growth-impacting mutations acquired during the media-adaptation period. It is important to note that the original Lenski strains show no significant growth rate differences between them under any of the temperatures studied, further supporting the claim that the *araA* and *recD* mutations differentiating the two Lenski strains is neutral under a wide range of environmental conditions.

The media-adapted 606P population acquired several mutations in genes whose biological function could explain the added growth benefit in the glucose media. Specifically, a large deletion in the *rbs* operon, involved in ribose metabolism and uptake, reached fixation in the population. Deletions in the *rbs* operon previously are reported to evolve in laboratory evolution experiments employing glucose minimal media with a high degree of parallelism (Phillips and Wilson, 2016; Tenaillon et al., 2016) due to the combination of being selectively advantageous and having a high mutation rate mediated by the nearby IS transposable element (Cooper et al., 2001). Another mutation that reached fixation involves a non-synonymous substitution affecting the translation initiation factor, protein chaperone, and cold-shock gene *infB*. Mutations in *infB* are observed in similar experiments (Barrick et al., 2009). Additionally, since *infB* is a cold-shock gene (Jones and Inouye, 1994; Gualerzi et al., 2003; Barria et al., 2013; Brandi et al., 2019), this may explain why 606P has a statistically significant increase in growth rate at 15°C compared to 607P (see Supplementary Figure 2). At a high frequency in the population but not reaching fixation, another IS mediated large deletion was present that impacts a total of eight genes, including the *cybB* gene coding for a cytochrome that detoxifies superoxide, *hokB, mokB* gene components of a toxin antitoxin system, *ydcA, ydcI, ydcJ* transcription regulator genes related to stress (Solomon et al., 2014), *trg* ribose chemotaxis related gene, and *ydcG* glucan synthesis gene. Deletions surrounding *hokB/mokB* mediated by IS elements are reported to occur in Lenski's LTEE strains and in mutation accumulation experiment (MAE) strains indicating the presence of a mutational hotspot (Woods et al., 2006; Tenaillon et al., 2016). Finally, a low frequency non-synonymous mutation in the gene *uxaB* involved in carbohydrate metabolism was also observed in our 606P population.

The media-adapted 607P population acquired fewer mutations, with none of them reaching fixation. A mutation reaching higher frequency was a deletion of Δ122 bp affecting the gene *ECB_02621*, which encodes a hypothetical protein as yet uncharacterized. Additionally, a low frequency mutation was observed in the intergenic region between the genes *yhjY* and *tag*, which may relate to DNA repair mechanisms. In both of the 606P and 607P populations, parallel mutations in the gene *nadR* were observed, which are common in laboratory evolution experiments using these strains with glucose as a carbon source (Woods et al., 2006; Phillips and Wilson, 2016). Most of the genes mutated in these populations are observed in similar experiments using glucose media, which gives evidence that these media-adapted mutations provide a good baseline for some of the adaptive mutations to the culture media. Importantly, the mutational profiles that arose in the media-adapted strains were very different, which allowed us to test genetic background contingencies on evolution under fluctuating temperatures with various levels of periodicity.

### 2.2. Periodic Environmental Oscillations Favor the Evolution of Generalists

To investigate the evolutionary contingencies on different fluctuating environment regimes and genetic backgrounds, the media-adapted populations, 606P and 607P, serve as the starting populations that are evolved under three different temperature fluctuation regimes: (1) stochastic (which serves as a control and is referred to as “random”), (2) periodic and fast (referred to simply as “fast”), and (3) periodic and slow (referred to as “slow”) (see Figure 1). Each of these three environmental treatments are comprised of four 606P replicates and four 607P replicates, totaling to twenty-four evolving populations. From each of the 24 final populations, a single representative clone was isolated, sequenced, and phenotypically characterized. In order to evaluate the adaptation of each evolved population to the experimental temperatures, the growth rate of each representative clone was measured at 15 and 43°C relative to its corresponding starting strain's growth rate (606P or 607P, respectively). Relative growth rates were calculated as the dimensionless ratios of the specific growth rates of the evolved strains in each temperature condition compared to their respective starting strain specific growth rate (see section 4).

**Figure 2:**
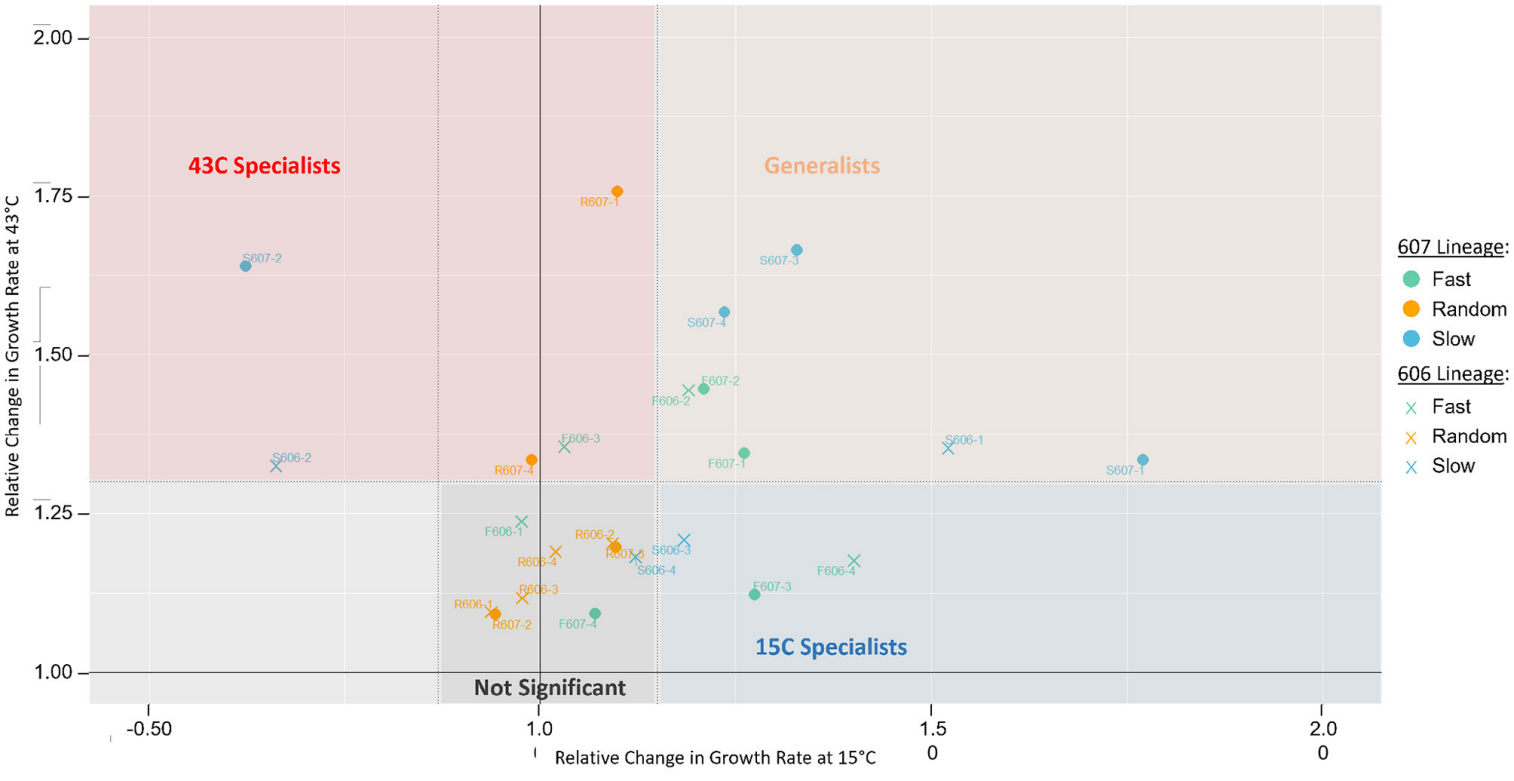
Growth rate changes of isolated clones from the final populations relative to their starting strain, 606P or 607P. The y-axis represents the relative change in growth rates of the evolved clones compared to their respective starting strain at 43°C. The x-axis represents the relative change in growth rates at 15°C. For each temperature, statistical significance between the clonal strains and their corresponding reference strain was assessed by a two-sample *T*-test with a pooled sample variance using all the strains at the same temperature. The dotted lines represent the statistically significant cut-offs, such that points falling outside the dotted lines have a *p*-value < 0.05. For these statistically significant strains, we classify them as either generalists or specialists. Namely, a clone is classified as a “generalist” if relative growth statistically significantly increased at both 15 and 43°C (in peach), classified as “43°C specialist” or “15C specialist” if relative growth increased in only 43°C (in red) or 15°C (in blue), respectively, and classified as “not significant” if relative growth did not increase in either temperature (in gray). Using a Fisher Exact Test, we find classification of generalist strains is significantly enriched in the slow and fast periodic strains compared to the random strains (*p*-value = 0.054). There is no significant environmental fluctuation treatment effect for specialists observed (periodic vs. random strains, *p*-value = 0.67). In support of periodic strains being more likely to be generalists, the periodic strains also have significant increased relative growth rate variation in both 15 and 43°C, suggesting periodic strains have a greater capacity to adapt to both temperatures. Whereas, the random strains have only increased variation at 43°C but not at 15°C. Using the F-statistic, we find periodic strains variance is significantly increased to that of the random strains at 15°C (*p*-value = 0.001).

In spite of the growth rate increase of 606P and 607P at the two temperature conditions, we observed further statistically significant growth rate increases for the final evolved clones with respect to these starting point populations in the fluctuating temperatures (see Figure 2). This observation demonstrated that the background mutations did not exhaust the adaptive potential to the 15 and 43°C conditions in our experiment. We classified evolved strains as “generalists” if there was statistically significant increase in relative growth rate in both temperatures. This is the least biased definition, avoiding applying an arbitrary threshold. An evolved strains was classified as a “specialist” to one of the temperatures if there was only significant growth rate increase at only one of the respective temperatures. Finally, strains with non-significant relative growth rates at both 15 and 43°C temperatures are considered “not-significant.”

The growth measurement data showed that many strains could adapt simultaneously to both the 15 and 43°C temperatures, demonstrating that generalists could evolve in our experiment (see Figure 2). The periodic regimes (fast and slow treatments) favored the evolution of a generalist strategy for both 606P and 607P lineages, as the periodic regimes had statistically significantly more strains for both lineages that evolved a generalist strategy compared to the random regimes (*p*-value = 0.054, see Figure 2). There was not a significant difference in the number of generalist strains evolved between the two periodic treatments (slow vs. fast) (*p*-value = 0.31). Moreover, no significant fluctuation regime effect for specialists was observed (periodic strains vs. random, *p*-value = 0.67). Importantly, a similar number of generations were spent in the 15 and 43°C conditions for all the final evolved strains except for the random R607 strains, which spent more generations in 43°C compared to 15°C (*p*-value < 0.01) (see Supplementary Figure 5). The increased number of generations the R607 strains spent at 43°C was not sufficient to drive the evolution of specialists to 43°C because only two out of the four R607 strains are classified as 43°C specialists, which is not significantly different from the R606 strains (*p*-value = 0.43). The random 606 evolved strains did not have a significant difference in the generations spent at 15 and 43°C (*p*-value = 0.23). Our evidence supported our hypothesis that the periodicity of the fast and slow fluctuation regimes was a major driving force for the evolution of generalists.

In support of periodic strains being more likely to be generalists, the periodic strains increased relative growth rate variation in both the 15 and 43°C conditions suggested a greater capacity to adapt to both temperatures. Whereas, the random strains had only increased variation at 43°C but *not* at 15°C. We performed the F-statistic to find that this difference in variance between the two periodic and random environmental regimes for the two temperatures was statistically significant (*p*-value = 0.0011). Note that two of the periodic slow strains had decreased relative growth rates at 15°C (S607-2 and S606-2). When these two strains were removed from the variance, periodic strains at 15°C still had significantly greater variance than the random evolved strains at this temperature (*F*-test, *p*-value = 0.03).

Interestingly, these two strains with decreased relative growth rates at 15°C (S606-2 and S607-2) internalized the slower temperature fluctuation regime, in the following sense: if the strains were cultured at 43°C during the acclimation step, then the evolved clone's growth rate at 15°C was significantly higher or equal to that of the respective starting strain (see Supplementary Figure 4); but if cultured at 15°C during the acclimation step, these two strains showed a significantly lower growth rate at 15°C than their respective starting strain's growth rate. This indicates that the strains depended on exposure to 43°C in order to have a higher growth rate when shifted to 15°C. Some of the mutations carried by these two strains that internalized the temperature fluctuation could have deleterious effects in 15°C, like the S607-2 deletion of the *fabF* gene, which is involved in lipid biosynthesis and the cold-shock response (Garwin et al., 1980). Additionally, according to our observations, mutations like a *fabF* deletion may be beneficial or neutral in the context of a previous exposure to the 43°C environment. This is indicative that the slow periodic environment had been internalized by these populations to some degree.

### 2.3. Mutational Analysis Demonstrates Parallel Evolution

**Figure 3:**
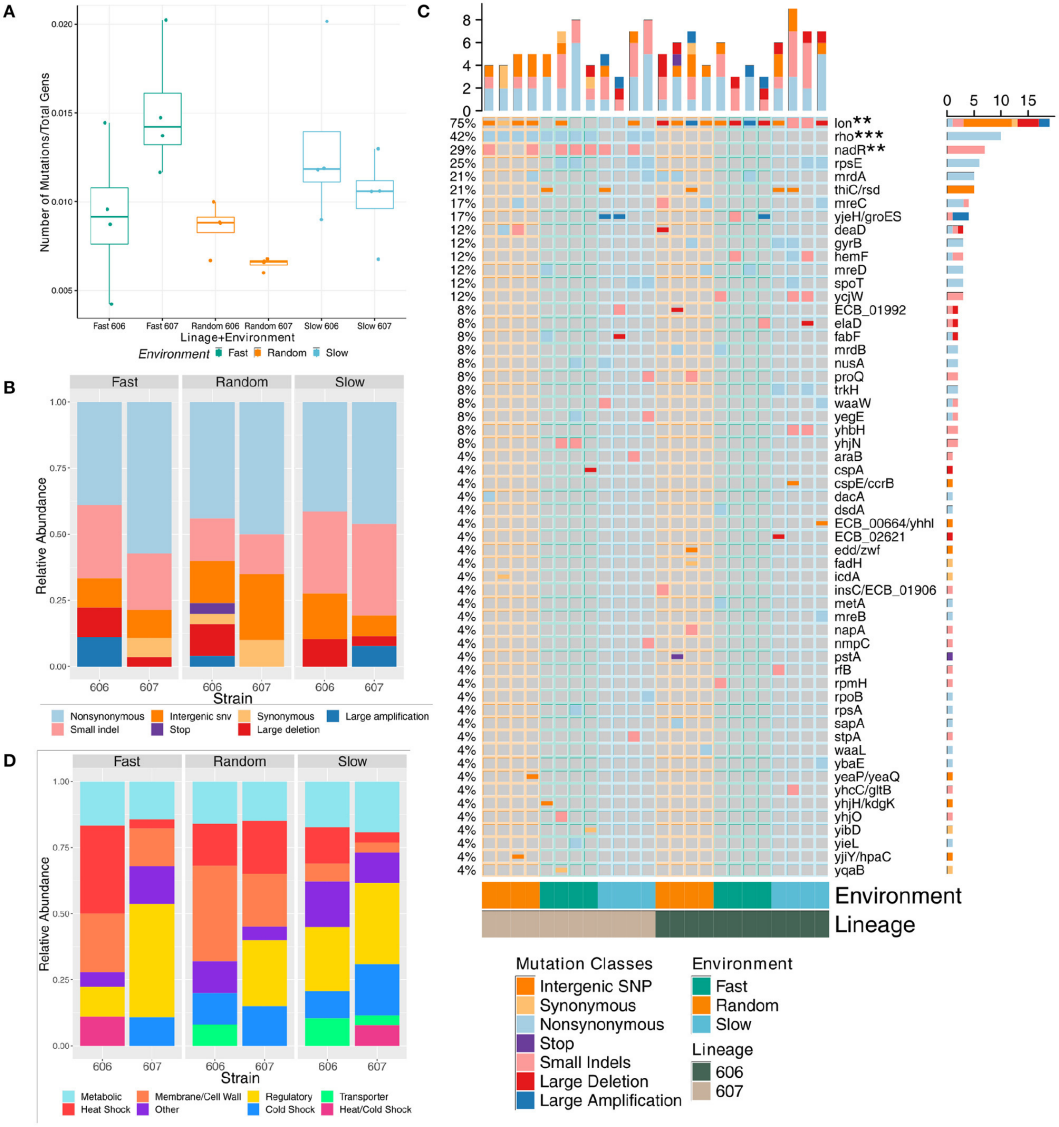
Mutational composition of the final evolved strains' population sequencing data evolved under fluctuating temperatures (note, mutations with frequency of 5% or greater in a final sequenced population are considered). **(A)** Boxplot showing the comparison of the total number of mutations normalized to total number of generations at the final time-point by strain and fluctuation regime. **(B)** Relative frequency of each mutation by class at the final time-point by strain and fluctuation regime. **(C)** Mutation incidence matrix of the population data. Mutations are coarse grained at the gene level. Large deletions and amplifications are counted once and collapsed to the repeatable gene. We use the Z-score to test significance of mutation enrichment between the evolved 606 lineage vs 607 lineage strains for genes that had mutations in three or more strains, and *nadR* and *rho* mutations were significantly enriched for the 607 lineage evolved strains (*p*-value = 0.017 with FDR = 0.08, and *p*-value = 0.00014 with FDR = 0.002, respectively). The mutation enrichment for periodic vs. random fluctuation regimes were assessed in the same manner with *deaD* enriched in random strains (*p*-value = 0.028, FDR = 0.0977) and *lon* enriched in periodic 606 evolved strains (*p*-value = 0.0015, FDR = 0.010). **(D)** Relative frequency of each mutation grouped by functional category at the final time-point by strain and fluctuation regime.

To investigate the molecular underpinnings of the changes in growth rates measured after our laboratory evolution step, genome sequencing of both the 24 resulting final populations (see Supplementary Tables 5–10 and Supplementary Figures 6–8) and the 24 representative clones (see Supplementary Tables 2–4) provided insight into the genes involved in the evolutionary response to each of the three temperature oscillation regimes under two different genetic backgrounds. Overall, mutations accumulated in the experimental populations and clones at about the same rate for each of the temperature fluctuation regime and genetic backgrounds when normalized to the total number of generations of each strain; only fast F607P populations were significantly different from both the random lineages (*p*-value = 0.031 and 0.018, respectively via *t*-test) (see Figure 3A) and no significant differences in the clonal strains (see Supplementary Figure 9A). Even though the number of generations is slightly greater for the random strains in both temperatures (see Supplementary Figure 5), the number of non-normalized mutations (i.e., total number of mutations) are not significantly different between any pairs of categories.

In a few cases, the the nature of the sequence changes was significantly different across environmental fluctuations and genetic background (see Figure 3B and Supplementary Figure 9B). Non-synonymous mutations and small indels were the most frequent types of mutations. Large amplifications and deletions mediated by transposable elements that could have dramatic adaptive consequences were also common. Using a one-sided Mann-Whitney *U*-test, we found that the evolved 606P strains had more large deletions than the 607 strains for both the populations and clones (*p*-value = 0.019 (average of 0.67 mutations per 606P population strain vs. 0.17 per 607 population strain) and = 0.021 (0.58 average large deletion mutations per 606 clonal strains vs. 0.17 per 607 clonal strains), respectively). For the environmental fluctuation regimes, random populations had *less* small indels than the periodic populations [*p*-value = 0.051 (0.88 average mutations per random vs. 1.81 per periodic)]. Interestingly, clonal strains classified as generalists had *more* small indels than the rest of the strains and specialists alone (*p*-value = 0.038 (2 average mutations per generalist vs. 1.12 per all other) and = 0.051 (2 average mutations per generalist vs. 1.12 per specialist), respectively), while the clonal strains that were classified as specialists had *more* non-synonymous mutations than the rest of the evolved clonal strains [*p*-value = 0.068 (1.5 average mutations per specialist vs. 0.94 per all other)]. Overall, there was some evidence to suggest that the environmental fluctuation regimes had an impact on the mutational aspects of the evolutionary process.

In our final populations and clones, there was a high degree of parallel evolution across the different temperature fluctuation regimes with many mutations impacting the same genomic region, and often the same gene (see Figure 3C and Supplementary Figure 9C). Two genomic regions were particularly affected, with mutated variants being observed in almost all of the final populations and clones, irrespective of the environmental regime: mutations in the *rho* ATP-dependent DNA-RNA helicase and mutations in the intergenic region of the *lon* heat shock protease. Using the Z-score to test significance of this mutation enrichment between the evolved 606P vs. 607P populations for these two genes, we found that indeed *rho* mutations were significantly enriched in the 607 lineage, along with *nadR* mutations [*p*-value = 0.00014 with false discovery rate (FDR) = 0.002, and *p*-value = 0.017 with FDR = 0.08, respectively]. Interestingly, despite mutations in *lon* gene in all the 606P evolved populations and many of the 607P populations, *lon* mutations are enriched in the two 606 lineage periodic strains (S606 and F606) (*p*-value = 0.0015, FDR = 0.010) because some periodic 606 populations had multiple *lon* mutations in a single population. This is reflected in the clonal data as well (see Supplementary Figure 9C). It is important to note that the parallelism was also present at the sequence level to a great extent in *rho* and in some instances of the intergenic region of *lon*.

The presence of *rho* and *lon* mutations, regardless of the temperature fluctuation regime, can be justified from the perspective of its importance for tolerating 43°C, as *lon* is involved in heat-shock response and elimination of stress-damaged proteins (Taylor, 1996). The presence of these mutations in the clones does not correlate with the relative growth rate results nor with the number of generations spent at 43°C, indicating that other accumulated mutations also impact the growth rates. Mutations in *rho* have also been consistently appearing with a high degree of repeatability in other adaptive evolution experiments of *E. coli* to high temperatures (Tenaillon et al., 2012), suggesting a crucial role of the termination factor in the 43°C condition. Additionally, in other gram-negative bacteria, *rho* transcription is significantly induced and associated with the RNA degradosome after exposure to cold temperatures, implicating that *rho* could be important in 15°C for our strains (Spaniol et al., 2013; Liu et al., 2014; Alvelos et al., 2017). Interestingly, clones of the strains evolved in the random environment have significantly *more* heat shock mutations than the periodic clones [*p*-value = 0.043 (0.88 average mutations per random vs. 0.50 per periodic)]. For the population data, strains classified as generalists had significantly *less* heat shock mutations than the other strains [*p*-value = 0.078 (0.57 average mutations per generalists vs. 0.94 per all other)].

There was a lesser degree of parallelism across the two lineages with only *deaD* enriched in evolved random populations (*p*-value = 0.028 with FDR = 0.0977, using the Z-score to test mutational enrichment between treatments for genes that had mutations in 3 or more strains) and lon enriched in periodic 606P evolved strains (see Figure 3C and Supplementary Figure 9C). This result provides evidence that evolution in our experiment is contingent upon genetic background. Importantly, the 606P final populations and clones lacked mutations in the *rho* gene, indicating that the adaptive benefit of these mutations was strongly contingent on the genetic background. Thus, the differences in genetic background of the starting populations, 606P and 607P, conditioned the distinct evolutionary trajectories of the 606P and 607P lineages seen in our experiments, most notably with the *rho* gene. Moreover, the evolved 607P lineage populations had *more* mutations in regulatory and cold shock genes than the evolved 606P lineage populations [*p*-value = 0.021 (2.08 average mutations per 607 populations vs. 0.75 per 606 populations) and 0.098 (0.92 average mutations per 607 populations vs. 0.50 per 606 populations), respectively], which is also reflected in the clonal data as well [*p*-value = 0.012 (1.0 average mutations per 607 clones vs. 0.33 per 606 clones) and = 0.035 (0.42 average mutations per 607 clones vs. 0.08 per 606 clones), respectively]. The final 606P populations had *more* mutations in heat shock and transporter genes than the final 607P populations [*p*-value = 0.0020 (1.17 average mutations per 606 vs. 0.50 per 607) and 0.071 (0.42 average mutations per 606 vs. 0.08 per 607), respectively], which is also reflected in the clonal data for the heat shock gene mutations [*p*-value = 0.0020 (0.92 average mutations per 606 clones vs. 0.33 per 607 clones); see Figure 3D and Supplementary Figure 9D].

Most of the remaining mutated gene variants were present in more than one replicate population and clone, but not to the extent of *lon* or *rho* observed parallelism (see Figure 3C and Supplementary Figure 9C). Some of these mutated genes are associated with a particular environmental fluctuation regime irrespective of lineage. While 607P strains had the largest number of mutations in regulatory genes, and random fluctuation regime strains had *less* regulatory than the periodic strains [*p*-value = 0.094 (0.62 average mutations per random vs. 1.81 per all other)]. Moreover, populations classified as not significant in Figure 2 also had significantly *less* mutations in regulatory genes than generalists and specialists combined [*p*-value = 0.063 (0.56 average mutations per not significant vs. 1.93 per all other)]. The two periodic treatments, slow and fast, are linked to mutations in the regulatory genes: *rpsE, ycjW, yhbH* (see Figure 3D in yellow and Supplementary Figure 9D in yellow). The regulatory, ribosomal protein gene *rpsE* was also mutated with high parallelism across the two lineages and the two periodic regimes, with variants affecting the same amino acid (see Figures 3C,D and Supplementary Figures 9C,D). In fact, in the relative growth rate analysis above, the strains evolved in the fast and slow fluctuation regimes had increased relative growth rates in the 15°C temperature compared to the strains evolved in the random treatment (see Figure 2).

Mutations in genes associated with cold-shock response: *cspA, cspE, deaD, fabF, gyrB, nusA*, and *proQ* genes, appeared predominantly in the 607P lineage strains irrespective of the temperature fluctuation regime (*p*-value = 0.098 for populations and = 0.035 for clones; see Figure 3D and Supplementary Figure 9D) (Jones and Inouye, 1994; Gualerzi et al., 2003; Barria et al., 2013). Though not significant, this could be reflected in the relative growth rate results since 6 out of 12 607P final clones have increased relative growth rate at 15°C compared to only 4 606P final clones despite both lineages spending a similar number of generations at 15°C (see Figure 2). This difference in relative growth rates at 15°C between the two lineages could be due to 606P starting population having a mutation in the cold-shock *infB* gene and increased growth at 15°C compared 607P starting strain (see Supplementary Figure 2). Additionally, S607-2 and F607-4 clones had large deletions in *fabF* and *cspA* cold-shock genes, respectively, and either decrease or no change in their relative growth rate at 15°C, respectively. The *nusA* non-synonymous substitutions were also related to 607P lineage mutations in the termination gene *rho*, as the *nusA* gene encodes a transcription termination/anti-termination factor and cold-shock element (Li et al., 2013). It was not surprising that only the 607P lineage final populations have mutations in *nusA* as only these populations had mutations in *rho*. Interestingly, the clone with both *nusA* and *rho* mutations, S607-1, had the highest relative growth rate at 15°C.

Mutations in genes associated with cell envelope, such as *mrd* and *mre* genes, appeared predominantly in the random environmental regime. This is reflected in the significance testing results since random population strains had *more* membrane and cell wall mutations than the evolved periodic strains [*p*-value = 0.045 (1.62 average mutations per random vs. 0.69 per periodic)]. Also, strains classified as not significant in Figure 2 had *more* membrane and cell wall mutations than generalists and specialists combined [*p*-value = 0.060 (1.56 average mutations per not significant vs. 0.67 per all other)]. Most of these cell envelope related genes have also been observed to mutate in laboratory evolution experiments using glucose media (Tenaillon et al., 2016), so this could indicate further adaptation to the media rather than to our temperature regimes. Similarly, there were mutations in several metabolic genes, such as thiamine synthesis gene *thiC* and *spoT*, which are related to adaptation to glucose media rather than the temperature conditions (Phillips and Wilson, 2016; Tenaillon et al., 2016).

Other mutations are present at high frequencies in some of the final strains. A salient set of these mutations affect heat-shock proteins other than the *lon* gene, like the chaperonin genes *groEL-ES*. Chaperonins *groEL-ES* are also essential and govern growth at low temperatures (Fayet et al., 1989; Ferrer et al., 2003). Therefore, we categorized the *groEL-ES* gene as both a heat and cold shock gene (see Figure 3D and Supplementary Figure 9D). Random populations and clones had significantly *less* mutations in genes that have both heat and cold shock functions than the two periodic treatments [*p*-value = 0.071 (0 average mutations per random vs. 0.25 per periodic populations) and = 0.045 (0 average mutations for random vs. periodic clones), respectively]. Moreover, strains classified as not significant in Figure 2 had significantly *less* of these cold and heat shock mutations than generalists and specialists combined for both the populations and clones [*p*-value = 0.053 (0 average mutations per not significant vs. 0.27 per all other populations) and 0.031 (0 average mutations per not significant vs. 0.33 per all other clones), respectively]. Two ways of potentially increasing chaperonin protein expression arose in four different populations (F606-2, F606-4, S607-1, and S607-2), none of which are classified as *not* significant in Figure 2. One way was through large genomic region amplifications and the other through a specific deletion in the regulatory region of the *groEL-ES* genes. The large genomic amplifications are not contingent on the genetic background (occurring in F606-4, S607-1, and S607-2) and affect a large number of proteins involved in both the heat- and cold-shock response, potentially having a great adaptive value for these populations. In fact, all the clonal strains with this large amplification of the *groEL-ES* gene have increased relative growth rates at 15 and 43°C, with the exception of S607-2 in 15°C, likely due to a large deletion of the cold-shock gene *fabF* and F606-4 in 43°C. Additionally, a similar small intergenic region deletion in the *groEL-ES* chaperonin genes present in the F606-2 population has been previously reported in high temperature adaptation (Yama et al., 2015), but may be useful for adapting to both hot and cold temperatures as this clone has increased relative growth rate in both temperatures (see Figure 2). Most of the large deletions and amplifications were mediated by mobile elements, which gives credence to these regions playing a large role in increasing the adaptability of the strains with these mutations.

Since strains evolved under the two periodic regimes had significantly more “heat and cold shock” genes mutated than strains evolved in the random regime for both the populations and clones (see above for *p*-values) and these strains had a significant enrichment for generalist strategy compared to random regime strains as well (*p*-value = 0.054), this gives further credence to our observations that specialists and generalists evolved in our experiments to deal with our periodic environmental fluctuation regimes. Thus, the ability to adopt a generalist adaptive strategy during evolution may be contingent on the evolution of the molecular machinery. As mentioned above, mutations in heat shock, cold shock, and regulatory genes may be especially important in adjusting the stability of an evolved strain's proteome in the new environmental conditions to maintain/restore the internal cell physiology of the starting strains in the optimum environment of 37°C, a possibility which we investigate further in the section below. Overall, a wide variety of mutations emerged in our laboratory evolution experiment with a high degree of parallelism across the different environmental fluctuation regimes, even at the molecular level (like *rho* mutations). There was a lesser degree of parallelism with respect to the lineage (like *lon* mutations), providing evidence that evolutionary trajectories are contingent on the genetic background.

### 2.4. Evolution Under Fluctuating Environments Promotes a Restoration of the Phenotypic Signature of the Optimum at 37°C

**Figure 4:**
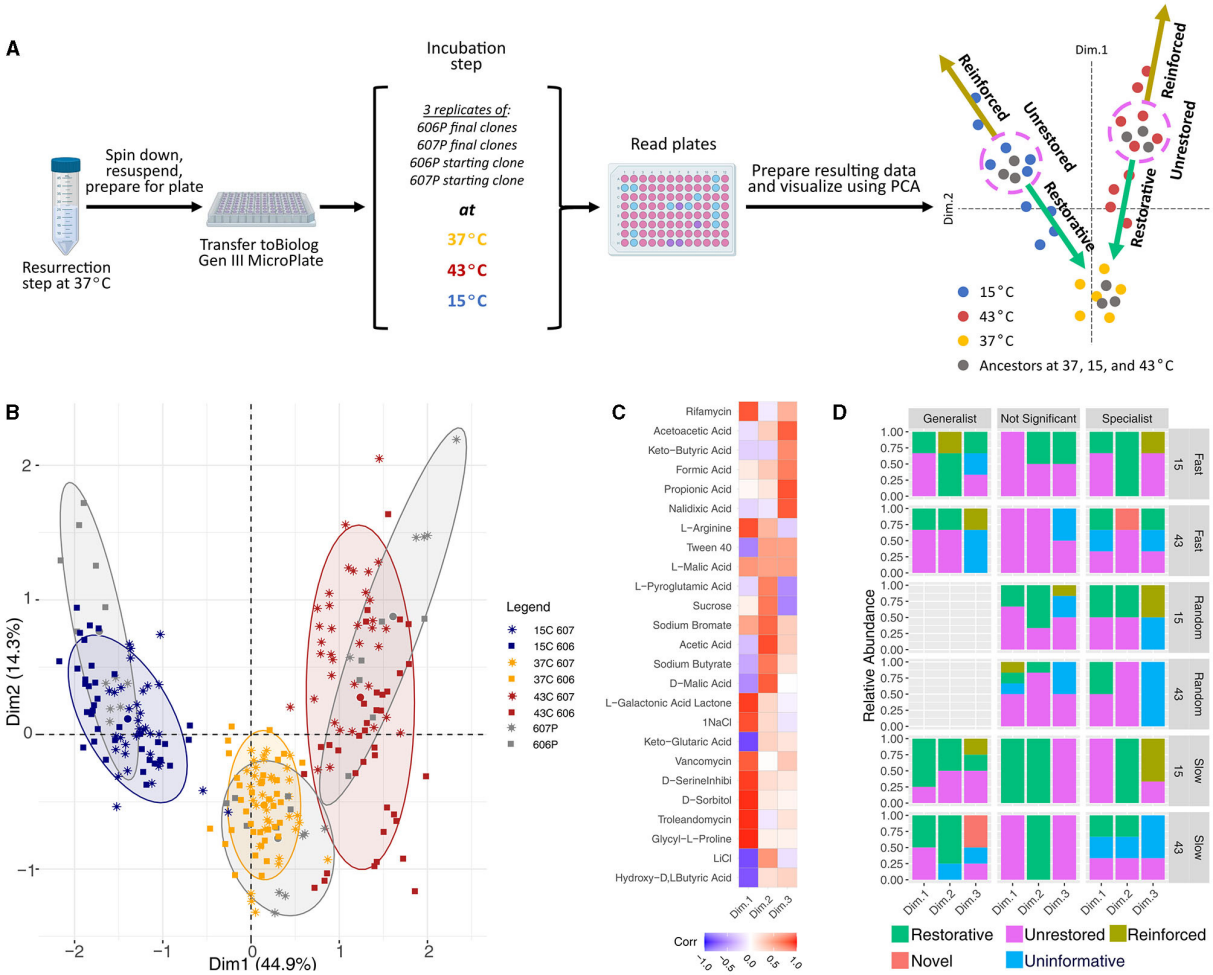
Biolog experimental schematic and data analysis results. **(A)** Diagram of the Biolog experiment in the three temperatures used in our laboratory evolution experiment. **(B)** Principal component analysis of the phenotypic fingerprint data. The colors indicate the temperature treatments of the Biolog plates (15, 37, and 43°C) and our reference starting strain's response at those temperatures. The larger dots mark the centroids of the clusters of our starting strains at each of the three temperatures. The axes shows the amount of variance explained by each of the components. PCA was obtained with the function *FactoMineR::PCA()*. PCA visulations employed *factoextra::fviz_pca_ind(…, addEllipses=TRUE, ellipse.level = 0.8)* with concentration ellipses that are drawn around each group using a multivariate normal distribution with the size of concentration ellipses as 0.8 in normal probability. **(C)** The correlations between the plate wells and the first 3 PCA dimensions, where the plate wells chosen were the top 10 wells that contributed to each of the three dimensions. **(D)** Restoration results for the Biolog data. Generalists, specialists, and not significant strains are shown. The results are also separated by the temperature condition the Biolog plates were grown at, and the experimental evolution environmental fluctuation treatment. The clones classified as generalists had *more* restorative and novel responses than specialists and non-significant strains combined (*p*-value = 0.030 and 0.075, respectively), and *more* restorative responses when compared to specialists alone when 15 and 43°C conditions were combined (*p*-value = 0.087). Generalist strains also had *more* novel and restorative responses than non-generalist strains at 43°C alone (*p*-value = 0.075 and 0.062), and have *less* unrestored responses than non-generalist strains at 43°C alone (*p*-value = 0.060).

The genes that were mutated in the laboratory evolution experiment primarily affect components of the proteostatic machinery, the translation machinery, and the transcription apparatus. The central role these processes play in determining the cellular state warranted a further exploration into the consequences of the acquired mutations on the phenotypic signature. Following a previous phenotype array method using Biolog plates (Hug and Gaut, 2015), the phenotypic signatures of the evolved representative clones and the media-adapted starting populations (606P and 607P) were measured at 15, 37, and 43°C (see Figure 4A). The phenotypes evaluated correspond to metabolic and chemical inhibition assays that our strains were not exposed to during our laboratory evolution experiment period. The hypothesis evaluated in Hug and Gaut (2015) puts forward the idea that the initial steps of adaptation to 43°C (the temperature which their strains were evolved during their laboratory evolution experiment) are the restoration of the cellular state in the environment the organism is previously adapted to, which is 37°C for *E. coli* strains.

Taking this a step forward, we evaluated if our more complex environment fluctuation regimes also exhibit a restorative response to the unstressed 37°C condition for both of the stressful temperatures (15 and 43°C) (see Figure 4A). We also investigated if: (1) periodic fluctuation regimes have different restorative responses to the unstressed state compared to the non-periodic control regime, and (2) evolved generalist vs. specialist adaptation strategies exhibit different restoration responses. This phenotypic evaluation also allowed us to study the consequences of the evolutionary adaptation to our temperature conditions in other regions of the ecological niche.

We first measured the baseline phenotypes of the 606P and 607P strains. Following Hug and Gaut (2015), we performed dimensionality reduction using Principal Component Analysis (PCA) on the measurements resulting from the phenotypic assays of the media-adapted strains to eliminate the effects of potential correlations between the phenotypes measured by the Biolog plates (which are not necessarily designed to obtain orthogonal phenotypes). The PCA of the media-adapted and evolved strains' phenotype array data shows that there were differences between the phenotypic signatures at the three temperatures, 15, 37, and 43°C (see Figure 4B), but not clear differences between the signatures for our two lineages. Three principal components were informative of the patterns in the media-adapted population data (see Supplementary Figure 10 and Figure 4C). In Figure 4B, the first principal component discriminates between the different temperatures, while the second principal component discriminates between the optimal temperature of 37°C and the other two stressful experimental temperatures.

As the media-adapted strains did not have distinct phenotypic signatures, the analysis of the evolved strains' phenotype array data was not separated by lineage. Consistent with the observed pattern for the media-adapted strains (in gray), clustering of the evolved clonal strains' phenotype array data showed that the primary discriminating factor in the phenotypic response of the evolved strains was the temperature their Biolog plate was grown in (see Figure 4B). Clustering by temperature fluctuation regime (treatment) or evolutionary trajectory assigned from relative growth results (strategy) did not occur. On the other hand, the cluster tree showed two primary splits corresponding to the 43°C temperature, and the 15 and 37°C temperatures. The 15 and 37°C temperatures then were split into two respective leaves for each temperature. This branching implied that the 37°C condition is more similar to the 15°C environment than to the 43°C environment with respect to the phenotypes assayed, and that the 43°C condition is more stressful as it appears to be furthest from the phenotypic response at the optimum (see Figure 4B). This branching is reflected in the results that the final evolved clones grown in the 15°C condition were more restorative to the optimum of 37°C than when those clones were grown in the 43°C condition (see Figure 4D). There is no correlation between the restoration results and the strength of the adaptative response to 43 and 15°C as measured by the relative growth rates (see Figure 2). This is discussed in more detail below. In general, the replicates tend to cluster together justifying taking averages in Hug and Gaut (2015) restoration analysis.

To assess the direction of the evolved phenotypic response, we used the classification and methodology described in Hug and Gaut (2015) (see Figure 4A). A strain at either the 15 or 43°C condition was considered restorative if partial restoration, restoration, or over-restoration occurred [following the same logic as (Hug and Gaut, 2015) to assign type of phenotypic movement]. We performed a Fishers Exact Test for each of the restoration response categories to compare between the Biolog plates grown at 15 vs. 43°C. We found that strains grown at 15°C had statistically significantly more restorative and reinforced responses, while less uninformative and novel responses than when the strains were grown at 43°C (*p*-value < 0.001, = 0.0045, < 0.001, and = 0.0036, respectively). The number of strains with unrestored responses is not significantly different between the two temperatures (*p*-value = 0.44).

To test if there were differences by lineage, fluctuation regime, or classified adaptive strategy for each restoration response type, we performed one-sided Mann Whitney U tests. For differences by lineage: clones evolved from the 606 lineage had *less* uniformative responses than the 607 lineage strains (*p*-value = 0.011) when 15 and 43°C conditions were combined. For the 43C condition alone, 606 lineage evolved strains had *more* reinforced and unrestored responses than 607 strains (*p*-value = 0.083 and 0.019, respectively), and had *less* uninformative responses than 607 strains (*p*-value = 0.0051).

For differences by environmental fluctuation regime: the clones evolved in the random environmental fluctuation regime had *less* restorative responses than the periodic strains when 15 and 43°C conditions are combined (*p*-value = 0.059). Random also had *less* uniformative responses in 15°C alone than the periodic treatment strains (*p*-value = 0.032).

For differences by classified evolutionary strategy in Figure 2: the clones classified as generalists had *more* restorative and novel responses than specialists and non-significant strains combined (*p*-value = 0.030 and 0.075, respectively), and *more* restorative responses when compared to specialists alone when 15 and 43°C conditions were combined (*p*-value = 0.087; see Figure 4D). Additionally, generalist strains had *more* novel and restorative responses than non-generalist strains at 43°C alone (*p*-value = 0.075 and 0.062), and have *less* unrestored responses than non-generalist strains at 43°C alone (*p*-value = 0.060). It is important to note that the number of generations in each temperature was not indicative of the amount of restoration and there was no significant difference in the generations between the two stressful temperatures and evolutionary strategies. The only defining feature that did correlate with restoration was the classification of generalists vs specialists. In total, these results support the hypothesis by Hug and Gaut (2015) that adaptation is toward the restoration of the cellular state at 37°C for *E. coli* strains since generalist strains have increased relative growth rate and increased variation at both 15 and 43°C, suggesting generalists have adapted more to these two conditions than the specialists and non-significant strains.

The evolutionary strategy adapted during our laboratory evolution period affected the amount of restoration the final evolved clone could obtain (see Figure 4D), while the genetic background, at least in our experiment, did not seem to play a role in restorative ability, except for uniformative and reinforced responses. Our Biolog phenotype assay data demonstrated that there was an overall tendency toward the restoration of the phenotypic signature of the respective starting strain in the optimal environment of 37°C, even in the face of a more complex adaptive challenge, like fluctuating environments.

## 3. Discussion

The goal of our laboratory evolution experiment was to better understand the adaptation of *E. coli* to various periodic fluctuation regimes vs. a non-periodic regime in the face of different genetic backgrounds, as measured by the adoption of specialist vs. generalist strategies, the mutational profiles that evolve, and the extent of phenotypic restoration. Firstly, we investigated the effect of the scale and periodicity of the environmental fluctuation regimes on the types of adaptation strategies that could evolve under these more complex environmental switching patterns by evolving two different lineages under two different periodic fluctuating temperature regimes with varying periodicity levels and under one non-periodic regime. By measuring the relative growth rate of the final evolved populations' representative clones relative to their starting population, we found that the evolution of generalists was favored in the periodic regimes and the evolution of specialists was favored in the random, control fluctuation regime that was non-periodic, irrespective of the number of generations spent at each temperature during evolution. Mutational analysis of the evolved populations and clones demonstrated that the different temperature fluctuation regimes did not completely constrain the molecular evolution, as there was parallelism across the different temperature fluctuation regimes with many mutations impacting the same genomic region, and often the same gene. Additionally, the mutational analysis hinted at the adaptive value of tuning the proteostatic machinery to the stressful temperature conditions, with mutations impacting heat- and cold-shock machinery, translation, and transcription. Thus, we hypothesized this stabilizing of the evolved strain's proteome in the new environmental conditions would promote the restoration of the internal cell physiology to that of the starting strains in the optimum environment of 37°C. Using a phenotypic array method characterized by Hug and Gaut (2015), we observed that the final evolved clones from both lineages showed a substantial amount of restoration to their respective starting strain's phenotypic response. Interestingly, evolved clones with generalist evolutionary strategies exhibited more restorative effects at both 15 and 43°C than specialists for the different environmental fluctuation treatments.

Secondly, we investigated the dependency of the evolutionary outcome on the genetic background. A striking outcome was that the original REL606 and REL607 Lenski strains we used for the media-adaption step and subsequent laboratory evolution step were identical except for neutral *ara* and *recD* mutations, yet resulted in highly consistent genotypic changes that were different, though addressing the same environmental conditions (37°C for media-adaptation step, and three fluctuating temperature regimes for our laboratory evolution step). Additionally, there was a lesser degree of parallelism with respect to lineage, providing evidence that evolution is contingent on genetic background, as seen in other experiments too (Woods et al., 2011; Barrick and Lenski, 2013; Achaz et al., 2014; Good et al., 2017). However, the genetic background, at least in our experiment, did not play a role in phenotypic restoration ability.

Our findings demonstrated that the scale and periodicity of environmental fluctuations play an important role in the specific ways laboratory organisms can adapt to selective pressures. Moreover, our phenotypic array results indicated an overall tendency toward the restoration of the phenotypic signature of the starting strain in the optimal environment, even in the face of a more complex adaptive challenge, like fluctuating environments. These results were not driven by differences in the number of generations spent in each temperature during the laboratory evolution experiment, as a similar number of generations was spent in both temperatures for all classes of strains except for the random 607P strains at 43°C. However, specialists and not significant strains evolved in this regime and lineage, which is similar to the results of the random 606 lineage that did not have a significant difference in the generations at 15 and 43°C, suggesting again, that the generational differences does not drive the preference for evolution in one temperature over the other, but rather the differences in the level of periodicity in our environmental fluctuation regimes is the major driving force.

The mutational observations, the relative growth rate gains in both environments (15 and 43°C) under the different temperature fluctuation regimes, and the impact of adaptation to such conditions on the phenotypic signature relative to the unstressed state can still be discussed further. Because of their high degree of repeatability, the mutation sets that evolve in our experiment are solid candidates for explaining the evolved clones' increases in growth observed in both of the stressful temperatures. Additional reconstruction experiments need to be carried out in order to unveil any potential epistatic interactions between the mutations. In addition, transcriptomic and proteomic analyses are needed to clarify the transcriptional and translational consequences of these mutations. Nevertheless, the potential consequences of the mutations can be discussed on the basis of our experimental observations and the current state of the literature.

In our experiment, the split between our two evolved lineages regarding the acquisition of a mutation in the termination factor *rho* can help clarify the molecular mechanisms that make *rho* mutants so pervasive in *E. coli* adaptive laboratory evolution experiments at high temperatures, and suggest that they should be expected to appear in experiments at low temperatures too. In this line of research, there is recent evidence that suggests the role of the mutations in *rho*, as observed in our experiment, is to restore the gene expression pattern of the unstressed condition (González-González et al., 2017). Yet, the distinct evolutionary trajectories observed for our two different lineages points toward an alternative explanation for the high parallelism of the *rho* mutations. Our results suggest that the background mutations of our starting strains conditioned the observed disjoint evolutionary trajectories. These include the *rbs* deletion, *infB* non-synonymous mutations, and large *cybB* deletion. The *rbs* deletion and the other low frequency mutations observed in our starting 606P strain affect metabolism or DNA repair, which makes them poor candidates for epistatically preventing the appearance of the *rho* mutations observed in our 607P final populations and clones (see Figure 1, Upper Panel and Supplementary Table 1).

However, there are two potential candidates in our starting 606P strain that could prevent *rho* mutations from occurring in our 606P final populations and clones. The first potential candidate is the mutation in *infB*, the translation initiation factor and and cold-shock gene. In the *E. coli* genome this gene is in the operon of *nusA*, an important gene component of *rho*-dependent termination and also a cold-shock response gene. Furthermore, *infB* has altered protein levels in *rho* inhibited cells (Cardinale et al., 2008). Thus, we hypothesize there may be a functional relationship between *infB* and *rho*, such that the *infB* background mutation in our starting 606P strain may have similar functional consequences to our observed *rho* mutations for both the hot and cold temperatures.

The second possible explanation is that the *cybB* deletion in our starting 606P strain might be sufficient to compensate for the potential growth rate benefit of the *rho* mutation in our 607P final populations and clones. This deletion contains the toxin/antitoxin genes, *hokB* and *mokB*. We conjecture that the prevalence of *rho* mutations in laboratory evolution experiments is related to the rho-dependent termination under normal conditions of genes with toxic effects on E.coli, like hokB and mokB (Cardinale et al., 2008). Therefore, in a stressful environment the termination abilities of *rho* could be impacted such that some of these toxic genes are suddenly expressed, which would have a fitness cost. We hypothesize that the *cybB* deletion observed in our starting 606P strain might have ablated some of these toxic elements potentially under the control of *rho*.

In either of these two cases, the mutational background of the starting point in laboratory evolution experiments must be taken seriously when considering the adaptive value of mutations in any laboratory evolution experiment. All adaptive laboratory experiments are contingent on the evolutionary history of the starting point reflected in its genomic architecture.

Another salient feature of our experimental results is that a significant fraction of the mutations affect components of the proteostatic machinery involved in the heat-shock response (Nonaka, 2006), some of which are also required for growth at low temperatures (Fayet et al., 1989; Ferrer et al., 2003). Some of these mutations occurred through large genome amplifications that presumably could lead to an increase in the expression of these proteins. The major genes affected were those of the chaperonin GroEL/ES and the heat shock protease Lon. Lon is an ATP-dependent protease that is induced upon heat shock. The Lon protease participates in cellular proteostasis by degrading misfolded proteins preventing aggregate formation (Rosen et al., 2002). This protease is also involved in some regulatory interactions as some of its clients are regulatory elements (Dopazo et al., 1987; Aertsen and Michiels, 2005; Van Melderen and Aertsen, 2009). Affecting the expression levels of *lon* will presumably have an effect on protein degradation. The complex relationships of the protein quality control system and its relative growth rate effects allow for the formulation of reasonable hypotheses for *lon* mutations observed in our experiment causing either an increase or decrease in expression (Bershtein et al., 2013; Cho et al., 2015). The temperature changes in our experiment may promote protein misfolding and the consequent accumulation of aggregates in the cytoplasm. An increased activity of *lon* could compensate for the permanent presence of these aggregates. On the other hand, a decrease in *lon* activity could also have an adaptive explanation in permanent stress conditions by allowing for the misfolded clients to be refolded by the other arm of the protein quality control system. It has been shown that defective *lon* mutants can rescue temperature sensitive *rpoD* mutants by increasing the amount of the sigma factor due to lower degradation in the high temperature conditions (Grossman et al., 1983). In order to adapt to constant high temperature stresses it may be preferred to lower the expression of the Lon protease in order to increase the amounts of other proteins whose activity has been reduced as a consequence of the temperature increase.

Many of the mutations observed in our final clonal strains could confer relative growth rate benefits at the cold temperature since many genes mutated are involved in the cold-shock response and/or are essential for growth at low temperatures. Despite the lack of studies for adaptation to our cold temperature condition (Dragosits and Mattanovich, 2013), there are many studies about the genes required for low temperatures and the cold-shock response (Fayet et al., 1989; Jones and Inouye, 1994; Ferrer et al., 2003; Gualerzi et al., 2003; Barria et al., 2013; Li et al., 2013). Drawing on these studies, we hypothesize that in addition to mutations in cold-shock genes, the mutations in ribosomal protein *rpsE*, chaperone protein *groEL-ES*, and the stress related transcriptional regulator *ycjW* are potential candidates for conferring relative growth rate benefits at cold temperatures given their high repeatability in our 606P and 607P final clones. Mutations in these three genes are not observed in adaptation experiments to optimal and hot temperatures, giving further credence to the hypothesis that they may play a role in increased growth at cold temperatures (Barrick et al., 2009; Tenaillon et al., 2012; Hug and Gaut, 2015). The many membrane and cell wall mutations observed could also play a role in adaptation to cold temperatures, but some of them have been observed in laboratory evolution experiments carried out at optimal temperature (Barrick et al., 2009). A way to address this issue would be to perform a laboratory evolution experiment at low temperatures in the spirit of the many examples of adaptation at high temperatures (Sandberg et al., 2014). This would also help overcome a limitation of our study, which is the difficultly in establishing the adaptive limits that each of our two temperatures impose on each other. For instance, although we observe in our experiment many *rho* mutants that are common in laboratory evolution experiments at high temperatures, we have only one instance of the *rpoB* mutations which are also frequently observed at such high temperature experiments. The absence of the *rpoB* mutations in our experiment could be explained by the observation that, while the *rho* I15N mutations are reported to increase the high temperature niche boundary in adaptive laboratory evolution experiments (Rodríguez-Verdugo et al., 2014), the *rpoB* mutations in contrast have been demonstrated to exhibit fitness trade-offs at lower temperatures. Thus, the *rpoB* mutations produce a niche shift rather than the niche expansion. Additionally, in other gram-negative bacteria, *rho* transcription is significantly induced and associated with the RNA degradosome after exposure to cold temperatures, suggesting that *rho* could be important for growth at colder temperatures, a potential further explanation for *rho* mutations being preferential in our experiment over *rpoB* mutations (Spaniol et al., 2013; Liu et al., 2014; Alvelos et al., 2017).

Another relevant feature of our experiment is the appearance of mutations that significantly lower the relative growth rate in the cold environment, like deletions in the cold-shock response *fabF, cspA*, and *deaD* genes. However, our results suggest that these same mutations can be neutral in that environment as long as there is a previous epoch of the hot temperature. Our results highlight that the complexity of the environmental treatment needs to be taken into account when aiming to draw general conclusions about the trade-offs set by the mutational basis of a particular adaptation strategy.

Lastly, our experiment considered for the first time the evaluation of the directionality of the phenotypic response of *E. coli* strains simultaneously evolved in two different environmental conditions. We observe that, predominantly, our evolved strains' responses to both temperatures move closer to the unperturbed state of its starting strain's response at 37°C. This restorative response is relevant in light of the potential limitations to adaptation that the dynamical nature of our experimental regimes poses. Measuring an array of phenotypes is a first step toward characterizing both evolutionary and ecological properties, such as niche breadth, of a particular environmental treatment. Using laboratory evolution experiments smartly, we can aim to better understand effects on niche breadth as driven by evolutionary contingencies.

Overall, our data reveal that the periodicity of the environmental fluctuation patterns affects the adaptation strategies that evolve. Furthermore, these adaptive strategies can be reached via different or parallel means of molecular evolution, and the genetic background can have an effect on what mutations can and cannot occur. However, no matter the underlying molecular evolution and adaptation strategy, a consistent adaptive strategy seems to be to stabilize the proteostatic machinery in both hot and cold temperatures by promoting the restoration of the internal cell physiology to that of the starting strains in the optimum environment of 37°C, which is especially seen for strains that evolved a generalist strategy. So there is an overall tendency toward the phenotypic restoration of the starting strain in the optimal environment, even in the face of a more complex adaptive challenge, like fluctuating environments.

## 4. Materials and Methods

### 4.1. Experimental Evolution

The first stage of the experiment involved a media-adaptation step for a month to the glucose minimal media with the two *E. coli* strains obtained from Lenski, REL606 and REL607, at 37°C (more details below). REL606 and REL607 only differ by two selectively neutral mutations present in REL607. The *araA* (D92G) mutation and a mutation in the exonuclease V gene *recD* (V10A) that has also been found to be neutral with respect to Lenski's original REL606 strain and present in the Lenski's long term experimental evolution (LTEE) strains (Phillips and Wilson, 2016; Tenaillon et al., 2016). The resulting media-adapted populations, which we call 606P and 607P, are used as the starting points for the laboratory evolution experiment under three different fluctuating temperature regimes between 15 and 43°C. Specifically, each of these populations was used to seed a laboratory evolution experiment with four replicates for each of the three environmental regimes for a total of 24 populations (see Figure 1). The environmental regimes consisted of three temperature switching regimes oscillating the culture temperature between 15 and 43°C. Two of these regimes encompassed periodic oscillations (termed fast and slow) while the third oscillated randomly serving as a control. After roughly 600 generations, the final populations were whole genome sequenced to detect potentially adaptive mutations. To characterize the outcome of the laboratory evolution period, single clones were isolated, whole genome sequenced and measurements of their growth rates and their phenotypic signatures were performed.

The experiment was seeded with isolated clones from *E. coli* REL606 and REL607 strains, which are derivatives of *E. coli* B, provided by the Lenski laboratory. The strains were chosen for their wide use in experimental evolution studies. The REL607 strain harbors a neutral marker that renders it unable to metabolize arabinoise (*Ara*^−^) and distinguishable from the REL606 strain in tetrazolium and arabinose (TA) plates. Clones were isolated from a single colony of the respective strain and then propagated daily in M9 minimal media (Difco) supplemented with glucose 4 *g*/*L* for one month at 37°C in order to adapt them to the experimental media conditions. These cultures were transferred by taking a 100μ*L* of culture and transferring to a new tube containing 20 *mL* of fresh media just before stationary phase was reached. From these two adapted starting cultures that harbored different mutational backgrounds from each other (see Supplementary Table 1), 24 tubes of fresh media were seeded with either the 606P or 607P starting culture in replicates of four for each of the three temperature fluctuation treatments (2 strains × 3 treatments × 4 replicates = 24) for a total of 12 seeded cultures for each of the two starting cultures. The three temperature fluctuation treatments were comprised of oscillations of the culture temperature between 15 and 43°C. The time for a culture tube to reach the desired temperature after a temperature switch was <35 min, and temperature acclimation to within two degrees of the target temperature was achieved in 25 min (see Supplementary Figure 1).

Of the three temperature fluctuation treatments, two were dependent on the optical density of the culture and the control one was random. The first optical density (OD) dependent treatment, termed “slow” or “S,” corresponded to a slow periodic fluctuating environment treatment that consisted of cycles in which the temperature was changed every time the strains reached an *OD*_830_ of 0.25, roughly half of their carrying capacity in our conditions, during each cycle. After this switch to the different temperature at an OD of 0.25, the strains were allowed to reach stationary phase and then were transferred to fresh media to begin a new slow treatment cycle. Thus, each cycle started after transferring to fresh media after the end of the previous cycle, and ended once the strains neared stationary phase. The second OD-dependent treatment, termed “fast” or “F,” corresponded to a faster periodic fluctuating environment treatment in which the temperature was switched at *OD*_830_ 0.15, 0.30, 0.45 in one cycle. After the final temperature switch at an OD of 0.45, the strains were transferred to fresh media to begin a new fast treatment cycle when the strains neared stationary growth phase. The random control treatment, termed “random” or “R,” sampled a random time for the culture to remain at a particular temperature from a uniform distribution between 3–5 h at 43°C and 5–15 h at 15°C. Since the growth rate at 15°C was slower, we had the strains spend a longer amount of time in this condition to ensure a similar number of generations was spent at 15 and 43°C during evolution. This also ensures that the random strains experience a similar number of temperature shifts as the periodic strains. Again, the strains were transferred to new media once they neared stationary growth phase to start a new random treatment cycle. In other words, by using the *OD*_830_ measurements, growth phase was closely monitored and the strains were consistently transferred near late exponential growth phase as the strains neared saturation at the end of each treatment cycle. Always transferring at the same point in the growth phase curves allowed selection responses to be due to the differences between the three fluctuating temperature regimes instead of due to the different positions in growth phase when transferred.

Since each of the three environmental fluctuation regimes consisted of four REL606 and four REL607 strains, it allowed us to follow the transfer schema in Lenski et al. (1991) by alternating between 606 and 607 strains, providing means for testing for cross-contamination. Every transfer was performed by taking a 100μ*L* of culture and transferring to a new tube containing 20 mL of fresh media. All cultures were incubated using a commercial batch culture BioSan LV Personal Bioreactor RTS1-C (Riga, Latvia), which controlled temperature to within 0.1°C. *OD*_830_ measurements were taken by the RTS-1C at 4 min intervals. The instruments allowed us to program our periodic fluctuations as a function of the optical density measurements and the random temperature fluctuation treatment as a function of time. All tubes were cultured at 2,000 rpm with 1 s reverse spinning intervals to ensure oxygenation.

### 4.2. Representative Clone Isolation

From both the starting (606P and 607P) and final (evolved) archived heterogeneous populations representative clones were isolated by picking single colonies on M9 glucose agar plates grown at 37°C. The picked clones were grown for a cycle in the experimental instruments at 37°C. The resulting 20 mL culture was aliquoted so half was immediately frozen in glycerol at −80°C and the other half was used for whole genome sequencing.

While there is a potential for large amounts of variation in the evolved populations, in our experiment we observed the domination of few population subsets on the basis of the high percentages of common accumulated mutations (see Supplementary Tables 5–10). We therefore chose the clone that is representative of each dominant subset in a population (covering almost the entirety of the evolved populations). Specifically, the representative clones were consistent with their parental populations' dominant mutations. The only exception was F607-1 clone, which had a discrepancy with its parental population in terms of the mutational composition (clone has amplification mutation in *groEL-ES* but population does not). The lower sequencing coverage of this particular population could explain this discrepancy as the clonal amplification could be a low frequency mutant missed in population.

### 4.3. Growth Characterization

To measure the growth rate of the representative clones from the evolved populations, we employed the optical density measurements provided by the RTS1-C instruments. The approach involved measuring the growth dynamics of replicate cultures from the representative clones after an acclimation period at the desired temperature.

Initially we performed competition assays, however after assaying few strains, the outcomes were heavily biased in recovering colonies from only the evolved strains. Thus, we decided to perform a phenotype measurement of the growth rate and other growth parameters of the strains independently. Since the exponential growth rate is a component of fitness in our experimental set up, as well as lag phase, carrying capacity and potentially other mechanisms like interference that could mediate competition, we used relative change in growth rate as a proxy quantitative measurement of adaptation. This measurement was experimentally more efficient and gave us granularity we were not obtaining using the competition assays.

In detail, the representative clones were resurrected at 37°C from the glycerol stocks into M9 media supplemented with glucose 4 *g*/*L*. After almost reaching saturation, they were acclimated to the tested temperature by transferring 100 μ*L* of the culture to 20 mL of fresh M9 media with glucose at the desired temperature. Then, halfway through the exponential phase, the cultures were transferred the same way to triplicates (or sometimes up to six replicates for the strains with greater variance) at the same tested temperature. Each of these experiments was done using the same media batch and in parallel with the appropriate reference starting strain either 606P or 607P depending on the representative clone measured.

To assess the effects of the pre-culture on the growth rate, we modified the above protocol to have two passages with replicates in the other temperature after acclimation to the target temperature. For example, if the acclimation passage was in 43°C, then we performed two additional passages at 15°C to monitor the previous effects at 43°C on growth at 15°C (see Supplementary Figure 4).

In order to determine the growth rate for each representative clonal strain, the optical density measurements were processed and analyzed with a custom script in R version 4.0.1. For each clonal strain assayed, the entire optical density time-series was fitted to a cubic spline using the function *stats::smooth.spline()* with a smoothing parameter modified accordingly to avoid overfitting in the presence of measurement noise. The corresponding curves were then passed to the function *stats::predict(…,deriv=1)* in order to obtain the derivative of the entire growth curve. The maximum value of the derivative was used as the estimate of the growth rate. This measurement was compared to the similar approach of *groFit::gcFitSpline()* (Kahm et al., 2010) and also to the resulting outcome of fitting the non-linear equation of the Gompertz model (Swinnen et al., 2004) to the optical density data using *stats::nls()* nonlinear (weighted) least-squares method. Both resulted in similar estimates. These calculated specific growth rates (in min^−1^) were summarized for each clonal strain to obtain the relative growth rate of each. Specifically, the relative growth rate for each clonal strain was calculated by taking the average of all the replicates divided by with the average of the corresponding reference ancestral strain providing a relative measurement accounting for variations in experimental conditions. Statistical significance between the test strain and the corresponding reference strain was assessed by a two-sample *T*-test with $t=\frac{\bar{x_{1}}-\bar{x_{0}}}{s_{p}\sqrt{\frac{1}{n_{1}}+\frac{1}{n_{0}}}}$, where s2 is pooled sample variance using all the ancestor and evolved strains at the same temperature, $\bar{x_{1}}$ and $\bar{x_{0}}$ are the sample means. This was done using measurements on their original scale. The alternative hypothesis was two-sided, that the two means are different. This testing was done using measurements on their original scale. The dotted lines in Figure 2 represent roughly the cut-offs corresponding to a *p*-value of 0.05 in the *t*-tests. All points falling outside of the dotted lines have a *p*-value < 0.05. The time window for the growth calculation was ~2,500 min (see Supplementary Figure 3). This figure supports that the duration of the growth calculation was appropriate.

### 4.4. Whole Genome Sequencing and Analysis

For the study of the evolutionary dynamics of our experimental populations, the genomic sequencing of the final populations and clones was conducted by directly pelleting the remaining culture after the transfer to fresh media. In doing so, we followed the recommendation of Sprouffske et al. (2016) that analyzed the effects of the archiving process on the mutational composition of heterogeneous bacterial populations to avoid bias introduced by selection acting on the freezing-thawing cycle. For the starting strains, 30 μ*L* of the glycerol stock were sequenced. All sequenced samples were treated as follows. The genomic DNA was isolated with the PureLink genomic DNA mini kit. The extracted genomic DNA was evaluated for quality using Nano-drop absorbance rations and quantified using Qubit dsDNA high sensitivity assay kit. All sequencing was done with 2 × 75 bp paired-end massively parallel sequencing on an Illumina MiSeq (San Diego, USA). Structural and single nucleotide changes at the genomic level were predicted from the Illumina reads using *breseq* (version 0.28.1) assuming that they pertained to an heterogeneous population (Bernstein and Carlson, 2014). The reads were aligned to an updated version of the *E. coli* REL606 reference genome file (GenBank accession NC 012967.1). All mutations reported were manually curated to ensure validity.

To better characterize the genomic composition of the starting populations, an additional sequencing round was performed using NexSeq. The DNA was extracted from 30 μ*L* of the glycerol stocks using the PureLink genomic DNA mini kit. We fragmented the DNA using 300 bp setting using Covaris. Libraries were prepared using KAPA LT DNA library prep kit with dual size selection to get the library size range between 200 and 400 bp. Libraries were quantified using Qubit and qualitated by Agilent bioanalyzer. Libraries were further quantified using KAPA QPCR and sequenced on Illumina NEXtseq500 as paired end 2 × 150 bp. The coverage of both populations averaged to 6,000. Of all the low frequency mutants detected and not reported in Supplementary Table 1 for readability, none of these were carried over in the laboratory evolution experiment.

R version 4.0.1 was used for plotting the mutational fingerprint (Gu et al., 2016).

### 4.5. Biolog Assays

Phenotypic assays for each of the 24 clones isolated from the final evolved populations and the two clones isolated from the ancestral populations (see section 4.2, representative clone isolation) were performed using Biolog Gen III plates (Biolog, Hayward, CA). These Biolog plates are 96 well plates comprised of 70 metabolic wells with one negative control well, and 22 chemical sensitivity wells with one positive control well. Protocols were adapted from the methods in Cooper and Lenski (2000) and Hug and Gaut (2015). Our strains were revived from glycerol stocks and incubated at 37°C for one growth cycle in fresh M9 minimal media supplemented with glucose 4 g/L. This amount of glucose was chosen to avoid starvation cycles. The strains were then transferred to fresh M9 media with glucose and grown again at 37°C. After reaching the middle of exponential growth phase as defined by an OD_830_ of approximately 0.25, the samples were spun down at 4,000 rpm for 10 min in order to form a cell pellet. The cell pellet was then washed in sterile PBS, and resuspended in 10 mL PBS by vortexing for 30 s. The solution was then spun down again at 4,000 rpm for 10 min, washed with PBS, and resuspended again in 20 mL of PBS. The resulting suspensions were aliquoted into 10 *mL* vials of Biolog Inoculating Fluid A. Bacteria were aliquoted in the inoculating fluid with the appropriate volume to obtain 95% transmittance, which was verified by spectrophotometry to within 3%. The equation *OD* = −*log*_10_(*T*), where *OD* is the *OD*_830_ and *T* is the transmittance, was used to calculate the dilution of the bacteria into the inoculating fluid.

After being aliquoted into the inoculating fluid, the bacteria and inoculating fluid mixture was vortexed for 20 s to ensure mixing, and then 100 μL of the inoculating fluid bacteria mixture was aliquoted into each of the 96 wells of the Biolog Gen III plates. After being plated, the plates were labeled and placed in incubators at the appropriate temperatures, either 15, 37, or 43°C, and then allowed to grow. Plates grown at 37°C were grown for 24–36 h, plates grown at 43°C were grown for 48–60 h, and plates grown at 15°C were grown for 72–96 h to ensure saturation of bacterial growth. After saturation, the plates were measured on a BioTek plate reader (BioTek, Winooski, VT) at 590 nm. The measurements were repeated over time to ensure the stability of the reading.

All Biolog plates were performed in technical triplicates for each of the 24 evolved clones at each of the three temperatures, for a total of 24 × 3 × 3 = 216 plates. The two starting strains were performed in six technical replicates for each of the three assayed temperatures in order to better assess the ancestral phenotypic response, for a total of 2 × 6 × 3 = 36 starting strain plates. In total, there are 216 + 36 = 252 biolog plates and points in the PCA plot (see Figure 4B).

### 4.6. Analysis of Biolog Data

The biolog optical density measurements (OD_590_) were processed following the instructions of the maker. First, normalization of each plate was performed by subtracting its negative control from all metabolic wells. Additionally, the chemical sensitivity assays were normalized for each plate by subtracting those wells from the value of the positive control. Both the negative and positive controls were removed from the analysis. Wells 86 and 94, which contain two different tetrazolium dyes (which can be interpreted as two additional positive growing conditions as no inhibitory effect from the dies was observed in any of the populations) had consistently higher values than the rest of the wells for all the evolved and starting strain samples but the values did not vary between the samples. Thus, these two wells were removed from our analysis. This left us with 92 phenotypic tests for each sample, represented as a vector in 92-dimensional space. An alternative data processing was also performed following (Hug and Gaut, 2015). The conclusions were robust to both data processing methods. Additionally, data quality control was done to correct for any technical replicates with some inconsistent well values, and thus a few samples were corrected by imputation by taking the mean well value of the remaining replicates. The resulting processed data was then centered. Clustering of the output of the Biolog data was performed using Ward's algorithm for hierarchical clustering with Euclidean distance.

In order to assess the direction of evolved strains' phenotypic responses with respect to their respective starting strain, we followed the methodology put forward by Hug and Gaut (2015). The analysis was performed on all the strains irrespective of lineage or environmental fluctuation treatment. The dimensionality reduction of the phenotypic measurements was performed using principal component analysis (PCA) on the processed data to remove correlated variables due to the Biolog plate design. The centroids of each set of starting replicates at the three different temperatures were calculated by k-means clustering. The first three principal components were selected according to the criterion in Peres-Neto et al. (2005) amounting to ~70% of the variance, and according to the informative principal components obtained from the talus plot (Henningsson et al., 2019). *T-*test hypothesis testing with family-wise error rate correction was conducted to determine the direction of the phenotypic response for each evolved strain and retained principal component in relation to the respective starting strain response at the optimal and the stressed temperatures. Following Hug and Gaut (2015), the responses were classified as restorative, partially restorative, over-restorative, reinforced, unrestored, novel, or uninformative, where restorative, partially restorative, and over-restorative were grouped into a restorative category for easier readability in figures.

All statistical analyses were conducted in R version 4.0.1. PCA was obtained with the function *FactoMineR::PCA()*. PCA visulations employed *factoextra::fviz_pca_ind(…, addEllipses=TRUE, ellipse.level = 0.8)* with concentration ellipses that are drawn around each group using a multivariate normal distribution with the size of concentration ellipses as 0.8 in normal probability. The code for the analysis of the paper as well as the growth and phenotypic data can be found in the following repository: https://github.com/AvivLab/Experimental-Evolution-Analysis.

## Data Availability Statement

The datasets presented in this study can be found in online repositories. The names of the repository/repositories and accession number(s) can be found at: https://www.ncbi.nlm.nih.gov/genbank/; https://www.ncbi.nlm.nih.gov/bioproject/756002; https://github.com/AvivLab/Experimental-Evolution-Analysis.

## Author Contributions

DB, XP-J, and AB conceived the project. DB, XP-J, and ML performed the experiment. ML, XP-J, and AB performed analysis and wrote the paper. KY, ML, and AB performed statistical analyses. AB supervised the project. ML was driver of the submission and edits. All authors contributed to the article and approved the submitted version.

## Funding

Support was provided by NIH CMBG training grant T32-GM007491 to ML, NIH MSTP training grant T32-GM007288 to DB, the Fulbright program to XP-J, and NIH R01-CA164468 and R01-DA033788 to AB.

## Conflict of Interest

The authors declare that the research was conducted in the absence of any commercial or financial relationships that could be construed as a potential conflict of interest.

## Publisher's Note

All claims expressed in this article are solely those of the authors and do not necessarily represent those of their affiliated organizations, or those of the publisher, the editors and the reviewers. Any product that may be evaluated in this article, or claim that may be made by its manufacturer, is not guaranteed or endorsed by the publisher.
