# Supplementary material for: Emerging Adaptive Strategies Under Temperature Fluctuations in a Laboratory Evolution Experiment of Escherichia Coli
Source: Front Microbiol. 2021 Oct 22;12:724982. doi: 10.3389/fmicb.2021.724982 (PMC8569431; doi:10.3389/fmicb.2021.724982)
Supplement: Supplementary file 1 [file Data_Sheet_1.pdf]

Supporting Information

Supplementary Figures, Tables, and Methods

Population Archive

Approximately every week, each evolving population was glycerol stocked. After transferring 100μL of the culture after each treatment cycle to fresh media, the remaining culture was centrifuged for 10 minutes at 4000 rpm to form a pellet, and the excess liquid was removed. The pellet was then re-suspended in 1 mL of fresh M9 media and 1 mL of glycerol (Sigma) 20% to a final glycerol concentration of 10%. The samples were then placed directly in a freezer at -80 °C for long term storage. Whenever the bacteria were glycerol stocked they were also cultured in tetrazolium and arabinose (TA) M9 agar plates at 37°C overnight checking for the correct colored phenotype to detect potential external and cross contamination, using the protocols of (Lenski et al., 1999).

Generations Elapsed during the Adaptive Evolution Experiments

The number of generations for each of the 24 evolved strains was calculated by transforming the optical density measurements, the absorbance units (au), into colony forming units (cfu). The transformation factor was derived by plate counting of the starting REL606 strain grown at 37°C in the RTS-1C with the same settings and media of the laboratory evolution experiment. The culture was sampled at mid exponential phase (OD $\approx$ 0.25) and plated in replicates of six serial dilutions in M9 agar with glucose. The colonies were counted after the overnight incubation at 37°C. With this method, we obtained the following average transformation factor:

$$\bar{\eta} = 1.74 \pm 0.1 \times 10^{10}\text{cfus/au} \tag{1}$$

The caveats of using this correction factor as opposed to a calibration curve is that it relies on extrapolation and ignores possible non-linearity's when approaching saturation. For our purposes, however, this method is sufficient as our cultures do not spend much time in stationary phase.

The cumulative number of cell divisions (CCD) was also calculated. The CCD provides an alternative measure of a meaningful time-scale for an adaptive evolution experiment and relies on the fact that most mutations occur at the moment of division (Lee et al., 2011). We calculated the number of generations and CCD for all of our populations by splitting the growth data by temperature and taking the initial and final optical density at each temperature segment. The following expressions were used to calculate the number of generations and the CCD:

$$G = \frac{1}{\log 2} \log\left(\frac{\bar{\eta}\text{OD}_f}{\bar{\eta}\text{OD}_0}\right) \tag{2}$$

And for all the m segments during the adaptive laboratory evolution:

$$CCD = \sum_{i=1}^{m} \bar{\eta}\text{OD}_0 \left(2^{G_i} - 1\right) \tag{3}$$

where OD_0 is the initial OD_{830} optical density and OD_f is the final OD_{830} optical density of the growth segment considered.

The number of generations and cumulative cell divisions were determined for each experimental population at each temperature (see Table S2). Overall the OD-dependent treatments did not differ significantly from the random treatment in either generations or CCD, as expected. They also did not show a significant asymmetry between the experimental temperatures. The populations with a random environment treatment went through slightly more generations due to the finite duration of the 15°C cycle, which

in turn could take place during a lag period not amounting to substantial growth. These populations also showed a marked asymmetry between the experimental temperatures in both generations and CCD indicating that they spent more generations in the 43°C. Nevertheless, it is important to note that the amount of generations or cell divisions spent in the 15°C environment is comparable for all treatments. The discrepancies in the total number of generations are explained by two instrumental problems. The first relates to some incomplete data as some of the instruments stopped recording the optical density measurements. The second, as a consequence of instrument malfunction we had to stop some of our populations earlier than others. These populations however did not show significantly fewer mutations suggesting that most of the adaptive mutations occurred in the early stages of the experiments.

Table S 1: Mutations found after the pre-adaptation period. In bold the two mutations present in the original REL607 strain.

Gene	606P Gene Frequency	607P Gene Frequency	Function	Mutation	Protein Change
**araA**	**0**	**100**	**L–arabinose isomerase**	**SNP T/C**	**D92G (GAC→GGC)**
**recD**	**0**	**100**	**Exonuclease V (RecBCD complex)**	**SNP T/C**	**V10A (GTT→GCT)**
nadR	100	0	Nicotinamidenucleotide adenylyltransferase	Coding +G at 4,616,183:1	Coding (655/1233 nt)
nadR	0	71.3	Nicotinamidenucleotide adenylyltransferase	SNP G/T	E367* stop
infB	100	0	Translation initiation factor IF2	SNP A/T	V85D
rbsD-[rbsR]	100	0	Multiple genes	IS150 mediated Δ 5,874bp at 3894997	Deleted rbsD, rbsA, rbsC, rbsB, rbsK, [rbsR]
yhjY/tag	0	20	Hypothetical protein/DNA glycosylase	Intergenic G/A	Intergenic (-42/-116)
uxaB	19.5	0	Tagaturonate reductase	SNP C/A	R429L
cybB-[ydcG]	75.4	0	Multiple genes	IS1 mediated Δ6,496bp at 1460927	Deleted cybB, ydcA, hokB, mokB, trg, ydcI, ydcJ, [ydcG]
ECB_02621	0	76.9	Conserved hypothetical protein	Δ 122 bp at 2,792,849	Deletion
ytfQ/ytfR	0	5.6	ABC sugar transporters	Intergenic A/C	Intergenic(+38/-102)

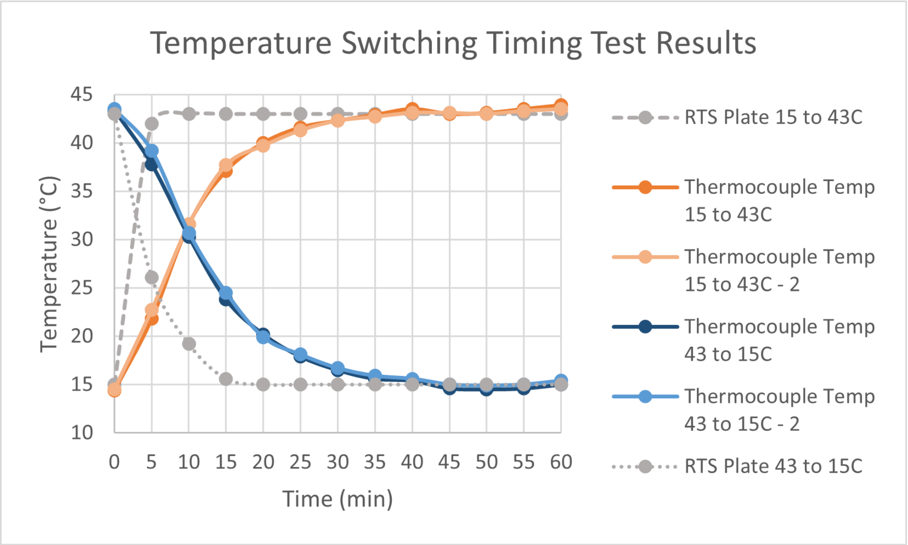

Figure S 1: Temperature switching timing test results for temperature fluctuation for both 43°C to 15°C and 15°C to 43°C. Results of experiment testing the transition timing. The transition between temperatures occurred in less than 35 minutes, which is less than one generation, thus suggesting that adaptation to the temperature transition period should not be a driving force in the evolution.

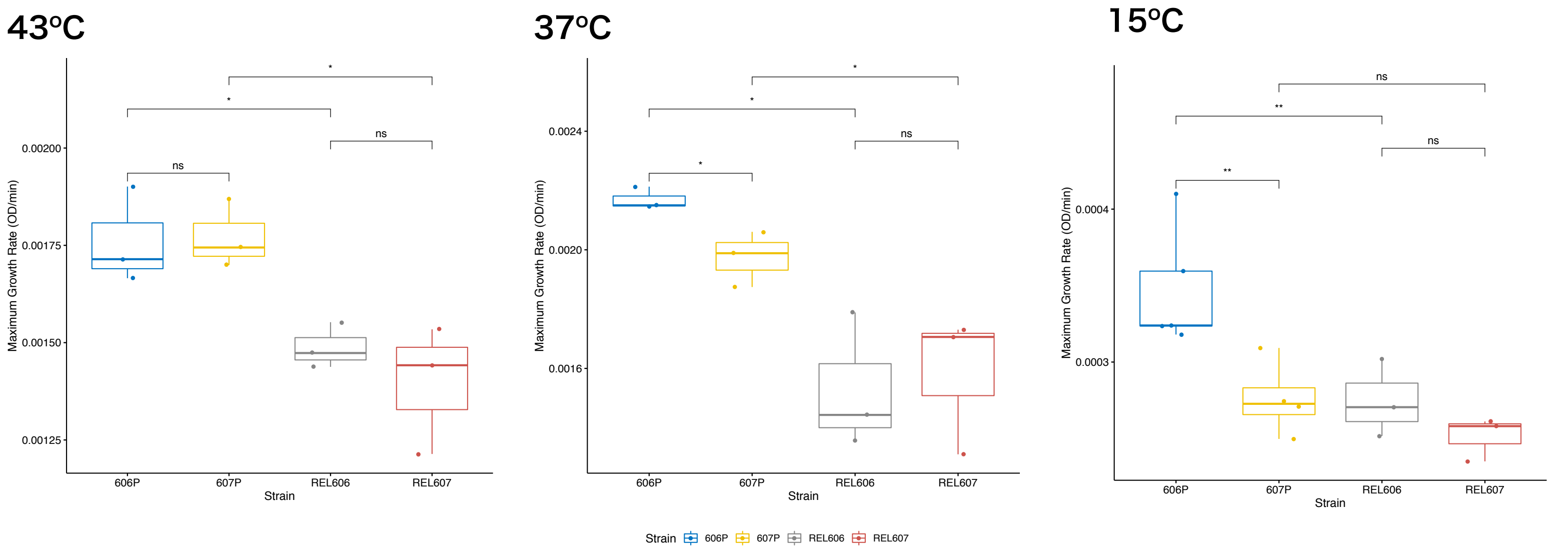

Figure S 2: Maximum growth rate comparisons of the pre-adapted and the Lenski strains at the temperatures of 43°C, 37°C and 15°C. The brackets indicate the groups being compared and are annotated with the outcome of the corresponding one-tailed two sample t-test with unequal variances (**ns**: not significant, *****: P-value < 0.05, ******: P-value < 0.01). P-values were corrected by False Discovery Rate.

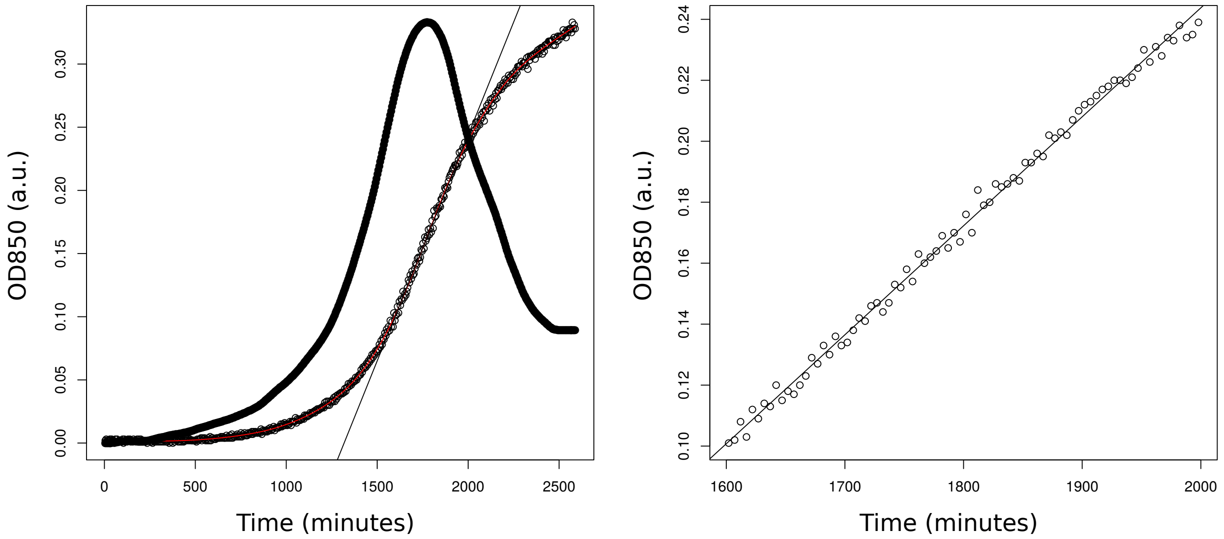

Figure S 3: Schematic of the methodology used for growth rate measurements: On the left, a sample plot of the optical density time-series of the starting strain 606P at 37°C. A cubic spline fit, in red, is shown on the OD trajectory. Accompanying, in darker dots is the derivative of the spline showing its maximum around the mid-point of the curve. In black solid line, linear regression is overlayed over the region when the derivative attains its maximum (that corresponds to the slope of this line). On the right, the inset of the region where the maximum growth rate is obtained with the linear regression overlayed as a solid line.

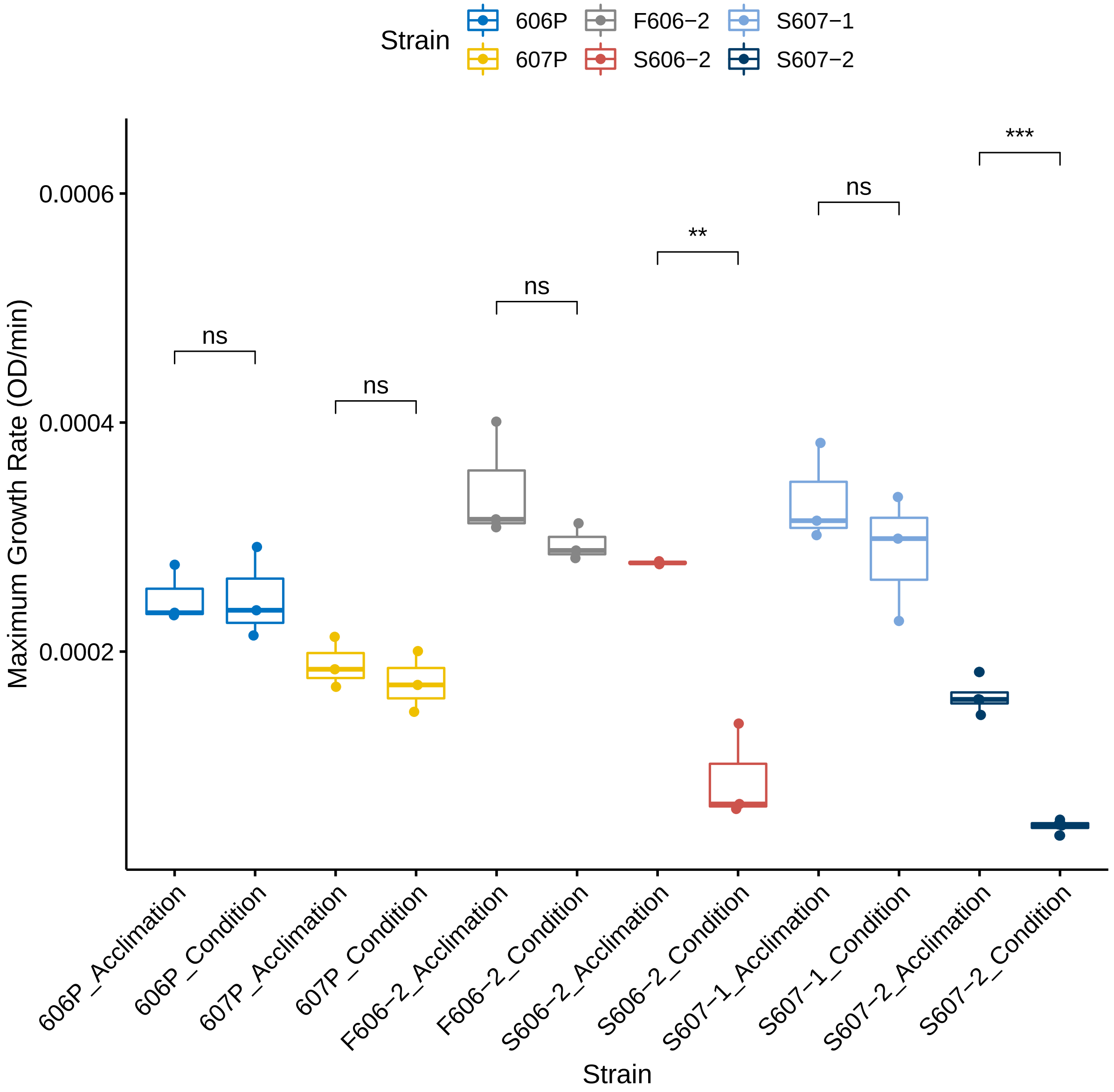

Figure S 4: Dependence of the relative growth rate at 15°C on the environment previously experienced. The acclimation labels identify the strains growing at 15°C for the first time after being revived from the glycerol stocks. The label condition, on the other hand, identifies the strains growing at 15°C after an acclimation step at the same temperature. The brackets indicate the groups being compared and are annotated with the outcome of the corresponding one-tailed two sample t-test with unequal variances (**ns**: not significant, ******: P-value < 0.01, *******: P-value < 0.001). P-values were corrected by False Discovery Rate.

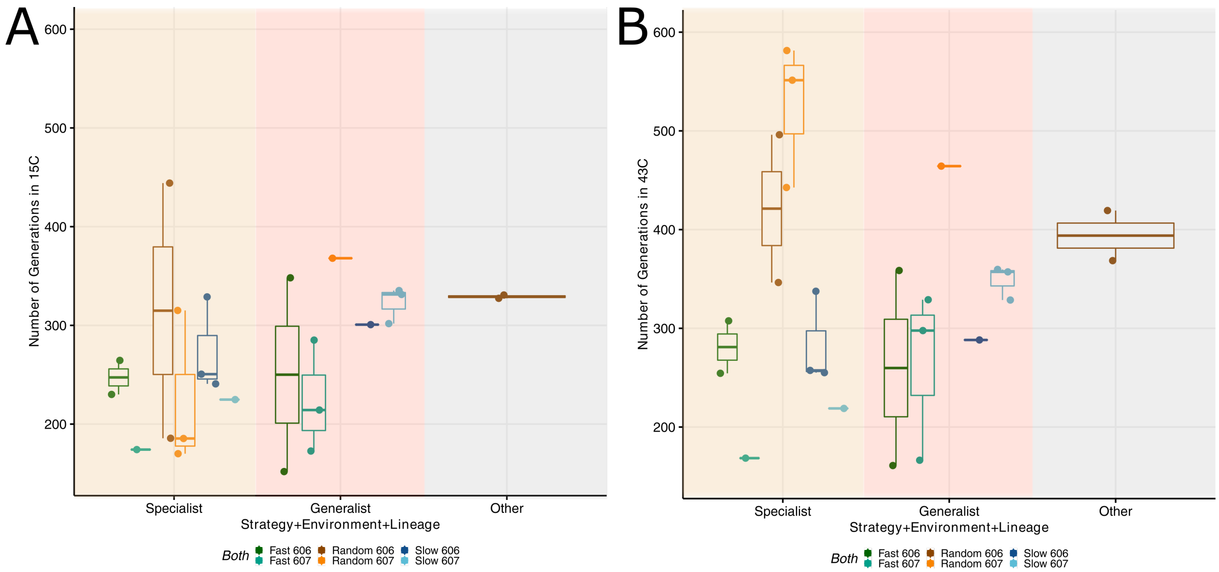

Figure S 5: Total Number of Generations of our Final Populations: Strains are separated by lineage with 606 strains having the darker color, and separated by evolution strategy with colors corresponding to those used in Figure 2. **A.** Generations spent at 15°C. **B.** Generations spent at 43°C.

Table S 2: Mutations found in the clones isolated from our final populations evolved in the random environmental treatment.

Genes	Function	Mutation Annotation	Strains
Ancestral:			
rbsD–[rbsR]	Ribose Metabolism	IS150-mediated Δ 5,874 bp at 3,894,997	R606-1,R606-2,R606-3,R606-4
cybB–[ydcG]	Multiple Genes	IS1-mediated Δ6,496 bp at 1,460,928	R606-1,R606-2,R606-4
infB	Translation	V85D (GTC→GAC)	R606-1,R606-2,R606-3,R606-4
nadR	NAD metabolsim	Coding (658/1233 nt) +G at 4,616,186	R606-1,R606-2,R606-3,R606-4
araA	Arabinose metabolism	D92G (GAC→GGC)	R607-1,R607-2,R607-3,R607-4
recD	Exonuclease	V10A (GTT→GCT)	R607-1,R607-2,R607-3,R607-4
yhjY/tag	Biofilm formation/DNA repair	Intergenic (-42/-116) G→A at 3,643,434	R607-1,R607-2,R607-4
nadR	NAD metabolism	E367* stop (GAG→TAG)	R607-3
ECB_02621	Unannotated	Coding Δ 122 bp at 2,792,849	R607-3
Regulatory:			
rho	Termination factor	I15N (ATC→AAC)	R607-2,R607-3,R607-4
rho	Termination factor	H295Y (CAT→TAT)	R607-1
polA/yihA	DNA Synthesis/Cell Division	Intergenic (+245/+135) Δ2 bp at 4,028,294	R606-1
Cold Shock:			
deaD	Ribosome Assembly	Coding (1223/1890 nt) +66bp at 3,241,872	R607-3
gyrB	DNA Coiling	R732H (CGC→CAC)	R606-3
proQ	RNA Chaperone	Coding (431435/699) nt Δ 5 bp	R607-2
proQ	RNA Chaperone	Coding (333-345/699 nt) Δ 13bp at 1,893,767	R606-3
Heat Shock:			
insL2/lon	Transposase/heat shock protease	Intergenic (-48/-108) $C \rightarrow T$ at 430,835	R607-3,R607-4
insL2/lon	Transposase/Heat shock protease	Intergenic (-92/-64) $G \rightarrow T$ at 430,879	R606-2,R606-4, R607-1
insL2/lon	transposase/heat shock protease	Intergenic IS mediated Δ1,350 bp at 429,504 +24 bp at 430,879	R606-1
insL2-[ppiD]	Multiple genes	Amplification at [430,618-434,631]	R606-3
Metabolism:			
nadR	NAD metabolsm	Coding (168/1233 nt) Δ1bp at 4,615,696	R607-2
nadR	NAD metabolsm	Coding (165/1233 nt) Δ1bp at 4,615,693	R607-1,R607-4
yjiY/hpaC	Membrane Protein/Reductase	Intergenic (-347/+46) T/A at 456,960	R607-3
edd/zwf	Glycolisis	Intergenic (-30/+205) A→C at 1,913,210	R606-3
thiC/rsd	Thiamine Biosynthesis/ Stationary Phase	Intergenic (-166/+68) A→T at 4,175,876	R606-3
pstA	Phosphate Uptake	W264* (TGG→TGA)	R606-2
napA	Nitrate Reductase	Coding (1510/2487 nt) Δ1bp at 2,251,232	R606-3
Cell Envelope:			
dacA	Cell Wall Synthesis	K76T (AAA→ACA)	R607-1
mrdA	Cell Wall Synthesis	Y397S (TAC→TCC)	R607-4
mrdA	Cell Wall Synthesis	V175I (GTC→ATC)	R606-2
mreC	Cell Shape Regulation	Coding (1077/1104 nt) +C at 3,326,793	R606-1
mreD	Cell Shape Regulation	P28L (CCG→CTG)	R606-4
Others:			
ogrK-[ECB 02013]	Multiple genes	Coding Δ 22,146 bp at 2,100,308	R606-2

Table S 3: Mutations found in the clones isolated from our final populations evolved in a fast, periodic environmental treatment.

Gene	Function	Mutation Annotation	Strains
Ancestral:			
rbsD–[rbsR]	Ribose Metabolism	IS150-mediated Δ 5,874 bp at 3,894,997	F606-1, F606-2, F606-3, F606-4
cybB–[ydcG]	Multiple Genes	IS1-mediated Δ6,496 bp at 1,460,928	F606-1, F606-2, F606-3, F606-4
infB	Translation	V85D (GTC→GAC)	F606-1, F606-2, F606-3, F606-4
nadR	NAD metabolsim	Coding (658/1233 nt) +G at 4,616,186	F606-1, F606-2, F606-3, F606-4
araA	Arabinose metabolism	D92G (GAC→GGC)	F607-1,F607-2,F607-3,F607-4
recD	Exonuclease	V10A (GTT→GCT)	F607-1,F607-2,F607-3,F607-4
yhjY/tag	Biofilm formation/DNA repair	Intergenic (-42/-116) G→A at 3,643,434	F607-2, F607-4
nadR	NAD metabolism	E367* stop (GAG→TAG)	F607-1,F607-3
ECB_02621	Unannotated	Coding Δ 122 bp at 2,792,849	F607-1,F607-3
Regulatory:			
elaD	Deubiquitinating protease	Coding (734/1212 nt) Δ 1 bp at 2,329,952	F606-4
rho	Termination factor	I15N (ATC→AAC)	F607-2,F607-4
rho	Termination factor	I15S (ATC→AGC)	F607-3
rpsE	Ribosomal Protein	G91S (GGT→AGT)	F607-3
rpsE	Ribosomal Protein	C112S (TGC→AGC)	F607-2
ycjW	Transcriptional Regulator	Coding (177-179/999 nt) IS150 (-) +3 bp	F606-1
yhbH	Ribosome Regulator	Coding (273/288 nt) +6bp at 3,281,680	F606-1
Heat Shock:			
insL2/lon	transposase/heat shock protease	Intergenic (-24/-132) G→A at 430,811	F606-1
insL2/lon	transposase/heat shock protease	Non-coding and intergenic (-68/-88) Δ1350 bp at 429,505	F606-2, F606-4
yjeH/groES	transporter/chaperonin	Intergenic (-200/-74) Δ3 bp at 4,349,333	F606-2
[insL-2]-[ppiD]	Multiple genes	Amplification at [430689-435601]	F606-3
[dsbD]-efp/ecnA	Multiple genes	Amplification at [4343517-4354989]	F606-4
yjdN-[insG]	Multiple genes	Amplification at [4304004-4485407]	F607-1
Cold Shock:			
amn/yeeN	AMP degradation/Chaperone Related	Intergenic (+260/-83) T→C at 1,994,774	F607-1
[yhjS]-hokA	Multiple genes	IS 150 meadiated Δ 23682 bp at 3,627,409	F607-4
Metabolism:			
yieL	Xylanase	W29R (TGG→CGG)	F607-3
hemF	Oxidase	Coding (657-683/900 nt) Δ27bp at 2,481,400	F606-2
kup/insJ-5	Potassium transporter/IS150 protein	Intergenic (+6/-50) +G at 3,893,551	F606-1
ygcQ	Flavoprotein	T161T (ACC→ACA)	F606-3
nadR	NAD metabolism	Coding (165/1233 nt) Δ1bp at 4,615,693	F607-2,F607-4
Cell Envelope:			
yfgA	Cell Wall Shape	Coding (841-877/1014 nt) Δ 37 bp at 2,562,787	F607-1
yhjN	Cellulose Synthase Regulator	Coding (2237-2245/2340 nt) Δ9bp at 3,619,855	F607-2,F607-3
mrdA	Cell Wall Synthesis	N428D (AAC→GAC)	F606-3
Others:			
ECB_01992	Unannotated	Amplification coding (185/216 nt)	F606-2

Table S 4: Mutations found in the clones isolated from our final populations evolved in a slow, periodic environmental treatment.

Gene	Function	Mutation Annotation	Strains
Ancestral:			
rbsD–[rbsR]	Ribose Metabolism	IS150-mediated Δ 5,874 bp at 3,894,997	S606-1, S606-2, S606-3, S606-4
cybB–[ydcG]	Multiple Genes	IS1-mediated Δ6,496 bp at 1,460,928	S606-1, S606-2, S606-3, S606-4
infB	Translation	V85D (GTC→GAC)	S606-1, S606-2, S606-3, S606-4
nadR	NAD metabolsim	Coding (658/1233 nt) +G at 4,616,186	S606-1, S606-2, S606-3, S606-4
araA	Arabinose metabolism	D92G (GAC→GGC)	S607-1,S607-2,S607-3,S607-4
recD	Exonuclease	V10A (GTT→GCT)	S607-1,S607-2,S607-3,S607-4
yhjY/tag	Biofilm formation/DNA repair	Intergenic (-42/-116) G→A at 3,643,434	S607-1,S607-3,S607-4
nadR	NAD metabolism	E367* stop (GAG→TAG)	S607-2
ECB_02621	Unannotated	Coding Δ 122 bp at 2,792,849	S607-2
Regulatory:			
rho	Termination factor	I15N (ATC→AAC)	S607-3,S607-4
rpsE	Ribosomal Protein	G91C (GGT→TGT)	S607-4
rpsE	Ribosomal Protein	G91A (GGT→GCT)	S606-4
Heat Shock:			
cadC/pheU-[yjeM]	Multiple Genes	Amplification at [4,341,178-4,362,612]	S607-1
[fxsA]-[yjeP]	Multiple Genes	Amplification at [4,347,401-4,366,500]	S607-2
insL2/lon	transposase/heat shock protease	Intergenic (-24/-32) G→A at 430,811	S607-3
insL2/lon	transposase/heat shock protease	Intergenic (-48/-108) $C \rightarrow T$ at 430,835	S606-1
insL2/lon	transposase/heat shock protease	Intergenic Δ1350bp at 429,505	S606-4
insL2/lon	transposase/heat shock protease	Intergenic (-69/-87) Δ1bp at 430,856	S606-3
Cold Shock:			
fabF-pabC	Cold Fatty Acid Synthesis	Coding Δ 650 bp at 1,167,222	S607-2
nusA	Elongation factor/cold shock inducible	T72A (ACC→GCC)	S607-1
Metabolism:			
ribC	riboflavin metabolism	F157L (TTT→TTG)	S607-2
hemF	Oxidase	Coding (621/900 nt) Δ 1 bp at 2,481,364	S607-2
hemF	Oxidase	W124R (TGG→AGG)	S606-2
prpE/codB	propionyl-CoA synthase/cystosine transporter	Intergenic (+198/-37) Δ 2bp at 327,440	S607-3
potA/pepT	ABC transporter/Peptidase	Intergenic (-132/-118) A→G at 1,201,096	S606-2
trkH	Potassium transporter	T20P (ACC→CCC)	S606-2
trkH	Potassium transporter	S349A (TCA→GCA)	S606-1
thiL	Thiamine Biosynthesis	L34F (CTC→TTC)	S606-3
nadR	NAD metabolism	Coding (165/1233 nt) Δ1bp at 4,615,693	S607-1,S607-3,S607-4
Cell Envelope:			
nmpC	Membrane Porin	Pseudogene (421/606 nt) T→G at 547,858	S607-4
ompF/asns	Membrane Porin/tRNA synthetase	Intergenic (-91/+411) 1 Δ bp at 1,004,265	S607-3
mreB	Cell Wall Synthesis	G117S (GGC→AGC)	S606-4
mreC	Cell Wall Synthesis	Coding (49/1104 nt) (CACCGCCAG)1→2 at 3,327,821	S606-2
Other:			
ECB_01992	Unannotated	Coding (154/216 nt) +CAGC at 2,103,887	S607-2
ECB_02621	Unannotated	Coding (645-705/849 nt) Δ61bp at 2,793,153	S606-1
elaC-yfbM	Multiple Genes	Coding Δ3382bp at 2,328,714	S606-3
elaD	Deubiquitinating protease	Coding (734/1212 nt)) Δ1bp at 2,329,959	S607-1
yegE	Motility	Coding (1456-1462/3306 nt) Δ 7 bp at 2,077,927	S607-4

Table S 5: Mutations found in our final REL606-derived populations evolved in the random environmental treatment. Only the mutations that are present in a frequency of more than 5% of the populations are shown.

Gene	R606-1	R606-2	R606-3	R606-4	Mutation Annotation
Ancestral					
rbsD–[rbsR]	100	100	100	100	IS150-mediated Δ 5,874 bp at 3,894,997
cybB–[ydcG]	100	100	0	100	IS1-mediated Δ6,496 bp at 1,460,928
infB	100	100	100	100	V85D (GTC→GAC)
nadR	100	100	100	100	Coding (658/1233 nt) +G at 4,616,186
Heat Shock:					
insL2/lon	72.4	0	0	0	Intergenic IS mediated Δ1,350 bp at 429,504 +24 bp at 430,879
insL2/lon	0	100	0	100	Intergenic (-92/-64) $G \to T$ at 430,879
insL2-[ppiD]	0	0	48.9	0	Amplification at [430,618-434,631]
Cold Shock:					
deaD	60.2	0	0	0	Coding Δ 142 bp at 3,241,224
Membrane and Cell Wall:					
mrdA	52.3	0	0	0	D312A (GAC→GCC)
mrdA	0	18.6/6.1	0	0	V175I (GTC→ATC)/D354A (GAC→GCC)
mrdB	0	65.2	0	0	S37N (AGC→ AAC)
mreC	33.3	0	0	0	Coding (1077/1104 nt) +C at 332,679
mreC	0	0	0	9.5/7.5	S141C (AGC→TGC)/R113S (CGT→AGT)
mreD	0	0	0	35/17	P28L (CCG→CTG)/ P36L
Transporter:					
sapA	0	59.1	0	0	Y187N (TAT→AAT)
pstA	0	12.3	0	0	W264 (TGG→TGA)
Metabolic:					
waaL	0	0	0	23.5	G101R
fadH	0	0	6.1	0	L216L
napA	0	0	100	0	Coding (1510/2487 nt) Δ1bp at 2,251,232
edd/zwf	0	0	100	0	Intergenic (-30/+205) A→C at 1,913,210
Others:					
gyrB	0	0	100	0	R732H (CGC→CAC)
thiC/rsd	0	0	100	0	Intergenic (-166/+68) A→T at 4,175,876
ogrK-[ECB 02013]	0	18.5	0	0	Δ 2,2146 bp at 2,100,308
proQ	0	0	100	0	Coding (333-345/699 nt) Δ 13bp at 1,893,767
insC/ECB_01906	8.3	0	0	0	Δ 7bp at 2,001,021 intergenic (641/+8)

Table S 6: Mutations found in our final REL607-derived populations evolved in the random environmental treatment. Only the mutations that are present in more than 5% of the populations are shown.

Gene	R607-1	R607-2	R607-3	R607-4	Mutation Annotation
Ancestral:					
araA	100	100	100	100	D92G (GAC→GGC)
recD	100	100	100	100	V10A (GTT→GCT)
yhjY/tag	100	38.0	0	100	Intergenic (-42/-116) G→A at 3,643,434
nadR	0	65.1	100	0	E367 stop (GAG→TAG)
ECB_02621	0	66.7	100	0	Coding Δ 122 bp at 2,792,849
Regulatory:					
rho	0	100	100	100	I15N (ATC→AAC)
rho	55.4/34.6	0	0	0	R221C (CGT→TGT) /H295Y (CAT→TAT)
Heat Shock:					
lon	0	13	0	0	E4E (GAG→GAA)
insL2/lon	100	0	0	0	Intergenic (-92/-64) $G \rightarrow T$ at 430,879
insL2/lon	0	0	100	100	Intergenic (-48/-108) $C \rightarrow T$ at 430,835
Cold Shock:					
deaD	0	53.3/7.4	0	0	D321A (GAT→GCT) /L40Q (CTG→CAG)
deaD	0	0	39.5	0	Coding (1223/1890 nt) +66bp at 3,241,872
Membrane and Cell Wall:					
dacA	100	0	0	0	K76T (AAA→ACA)
mrdA	0	0	0	100	Y397S (TAC→TCC)
mreC	0	0	8.8	0	L84Q (CTG→CAG)
yjiY/hpaC	0	0	94.2	0	Intergenic (-347/+46) T/A at 456,960
Metabolic:					
icdA	0	5.8	0	0	D158D GAC→GAT)
nadR	100	0	0	100	Coding (165/1233 nt) Δ1bp at 4,615,693
Others:					
yeaP/yeaQ	0	0	0	5.5	Intergenic (+34/+233) T/C at 1,856,003

Table S 7: Mutations found in our final REL606-derived populations evolved in the fast periodic environmental treatment. Only the mutations that are present in more than 5% of the populations are shown.

**Gene**	**F606-1**	**F606-2**	**F606-3**	**F606-4**	**Mutation**
Ancestral					
rbsD–[rbsR]	100	100	100	100	IS150-mediated Δ 5,874 bp at 3,894,997
cybB–[ydcG]	29.8	100	100	100	IS1-mediated Δ6,496 bp at 1,460,928
infB	100	100	100	100	V85D (GTC→GAC)
nadR	100	100	100	100	Coding (658/1233 nt) +G at 4,616,186
uxaB	61.4	0	0	0	R429L (CGT→CTT)
Regulatory:					
ycjW	12.5	0	0	0	IS150 (-) +3 bp coding (177-179/999 nt)
elaD	0	0	0	100	Δ 1 bp at 2,329,952 coding (734/1212 nt)
Heat Shock:					
yjeH/groES	0	100	0	0	Δ3 bp at 4,349,333 intergenic (-200/-74)
[insL-2]-[ppiD]	0	0	49.7	0	Amplification at [430689-435601]
[dsbD]-efp/ecnA (groES-EL)	0	0	0	62.7	Amplification at [4343517-4354989]
lon	0	0	6.7	0	D445E
insL2/lon	8.3	0	0	0	Intergenic (-24/-132) G→A at 430,811
insL2/lon	71.3	0	0	0	Intergenic (-48/-108) $C \to T$ at 430,835
insL2/lon	0	94.0	0	94.3	Δ1350 bp at 429505 non-coding and intergenic (-68/-88)
Metabolic:					
hemF	0	51.3	0	0	Δ27bp at 2,481,400 coding (657-683/900 nt)
dsdA	6.1	0	0	0	I265T (ATC→ACC)
metA	13.7	0	0	0	I124L (ATC→CTC)
Membrane and Cell Wall:					
mrdA	0	0	61.5	0	D354A (GAC→GCC)
mrdA	0	0	14.3	0	N428D (AAC→GAC)
mrdB	57.6	0	0	0	R208S (CGC→AGC)
mreD	0	0	5.8	0	V161A (GTG→GCG)
Others:					
rpmH	6	0	0	0	Amplification +CG at 3,844,715 coding

Table S 8: Mutations found in our final REL607-derived populations evolved in the fast periodic environmental treatment. Only the mutations that are present in more than 5% of the populations are shown.

Gene	F607-1	F607-2	F607-3	F607-4	Mutation
Ancestral:					
araA	100	100	100	100	D92G (GAC→GGC)
recD	100	100	100	100	V10A (GTT→GCT)
yhjY/tag	29	100	9.8	100	Intergenic (-42/-116) G→A at 3,643,434
nadR	42.9	11.2	41.9	0	E367* stop (GAG→TAG)
ECB_02621	63.3	0	43.2	0	Coding Δ 122 bp at 2,792,849
Regulatory:					
nusA	0	0	27.8	0	D466N (GAT→AAT)
rho	100	77.4	20.6	100	I15N (ATC→AAC)
rho	0	0	30.9	0	I15S (ATC→AGC)
rho	0	15.5	42.2	0	V11A (GTT→GCT)
rpsA	0	0	38.9	0	D160V (GAT→GTT)
rpsE	0	59.2	21.8	0	C112S (TGC→AGC)
rpsE	0	0	12.4	0	G91S (GGT→AGT)
yegE	0	0	22.1	0	T778P
Heat Shock:					
insL2/lon	0	14.3	0	0	Intergenic (-24/-132) G→A at 430,811
Cold Shock:					
fabF	56.5	0	0	0	I115N (ATC→AAC)
Membrane and Cell Wall:					
mreD	32	0	0	0	F34S
yhjN	0	60.5	7.3	0	Coding (2237-2245/2340 nt) Δ9bp at 3,619,855
yhjO	0	19.4	0	0	IS1(-) mediated +9bp coding (2015-2023/2619 nt) at 3,622,706
Metabolic:					
nadR	0	80.5	0	100	Coding (165/1233 nt) Δ1bp at 4,615,693
nadR	0	0	21.5	0	Coding (861/1233 nt) Δ2bp at 4,616,389
Others:					
thiC/rsd	50	0	0	0	Intergenic (-149/+85) T→A at 4,175,859
yhjH/kdgK	50.1	0	0	0	Intergenic (-153/-79) A→G at 3,608,832
yqaB	0	10.1	0	0	A70A (GCG→GCA)
yieL	0	0	10.4	0	W29R (TGG→CGG)
yibD	0	0	0	11.3	I192I (ATT→ATC)
[yhjS]-hokA	0	0	0	82.8	Coding IS150 mediated Δ 23,682 bp at 3,627,409

Table S 9: Mutations found in our final REL606-derived populations evolved in the slow periodic environment. Only the mutations that are present in more than 5% in the populations are shown.

Gene	S606-1	S606-2	S606-3	S606-4	Mutation Annotation
Ancestral					
rbsD–[rbsR]	100	100	100	100	IS150-mediated Δ 5,874 bp at 3,894,997
cybB–[ydcG]	83.8	82.3	100	100	IS1-mediated Δ6,496 bp at 1,460,928
infB	100	100	100	100	V85D (GTC→GAC)
nadR	100	100	100	100	Coding (658/1233 nt) +G at 4,616,186
Regulatory:					
rpsE	0	0	35.1	0	G91S (GGT→AGT)
rpsE	0	0	0	93.6	G91A (GGT→GCT)
ycjW	0	62.7	38.6	0	Coding IS150 (-) mediated +3bp at 1,382,152
yhbH	0	37.2	46.2	0	Coding (268/288 nt) +6bp at 3,281,675
Heat Shock:					
insL2/lon	0	0	25.8	0	Intergenic (-69/-87) Δ1bp at 430,856
insL2/lon	89.1	0	0	0	Intergenic (-48/-108) $C \to T$ at 430,835
insL2/lon	0	17.2	0	0	Intergenic (-70/-86) +40bp at 430,857
insL2/lon	0	0	0	97.5	Intergenic Δ1350bp at 429505
Cold Shock:					
cspE/ccrB	0	10.5	0	0	Intergenic (+40/+15) G→T at 640,122
Metabolic:					
hemF	0	21.8	0	0	W124R (TGG→AGG)
hemF	0	0	21.7	0	Coding (367-377/900 nt) Δ11bp at 2,481,110
yhcC/gltB	0	48.9	0	0	Intergenic (-66/-516) Δ1bp at 3,281,675
spoT	0	12.9	0	0	N208Y (AAC→TAC)
rfB	6.0	0	0	0	Coding (112/1086 nt) +T at 2,046,181
Transporters:					
trkH	0	21.5	0	0	T20P (ACC→CCC)
trkH	89.1	0	0	5.1	S349A (TCA→GCA)
ybaE	0	0	0	7.6	A257V
Membrane and Cell Wall:					
mreB	0	0	0	70	G117S (GGC→AGC)
mreC	0	0	0	8.7	H179R (CAC→CGC)
Others:					
[elaC]-yfbM	0	0	14.5	0	Coding Δ3382bp at 2,328,714
gyrB	7	31	0	0	R732H (CGC→CAC)
thiC/rsd	17.9	18.3	0	0	Intergenic (-166/+68) A→T at 4,175,876
ECB_02621	87.8	0	0	0	Coding (645-705/849 nt) Δ61bp at 2,793,153
ECB_00664/yhhl	0	0	0	7.1	Intergenic (+4/-329) T→A at 717,389
waaW	0	0	0	5.5	V268I (GTA→ATA)

Table S 10: Mutations found in our final REL607-derived populations evolved in the slow periodic environmental treatment. Only the mutations that are present in more than 5% of the populations are shown.

Gene	S607-1	S607-2	S607-3	S607-4	Mutation
Ancestral:					
araA	100	100	100	100	D92G (GAC→GGC)
recD	100	100	100	100	V10A (GTT→GCT)
yhjY/tag	100	0	100	52.6	Intergenic (-42/-116) G→A at 3,643,434
nadR	0	100	0	55.5	E367 stop (GAG→TAG)
ECB_02621	0	100	0	63.3	Coding Δ 122 bp at 2,792,849
Regulatory:					
rho	0	0	94.8	59.1	I15N (ATC→AAC)
rho	0	0	0	39.4	V11A (GTT→GCT)
rpoB	0	0	0	58.2	G907S (GGT→AGT)
rpsE	0	0	65	44.3	G91C (GGT→TGT)
nusA	46.6	0	0	0	T72N (ACC→AAC)
nusA	8.0	0	0	0	T72A (ACC→GCC)
nusA	30.0	0	0	0	W62R (TGG→CGG)
stpA	0	0	8.9	0	Coding (246-248/405 nt) Δ 3 bp at 2,693,001
yegE	0	0	0	48.7	Coding (1456-1462/3306 nt) Δ 7 bp at 2,077,927
Heat Shock:					
cadC/pheU-[yjeM] (groES-EL)	58	0	0	0	Amplification at [4,341,178-4,362,612]
[fxsA]-[yjeP] (groES-EL)	0	71.6	0	0	Amplification at [4,347,401-4,366,500]
insL2/lon	0	0	23.8	0	Intergenic (-24/-32) G→A at 430,811
Cold Shock:					
[fabF]-[pabC]	0	32.5	0	0	Coding Δ 650 bp at 1,167,222
Metabolic:					
spoT	0	0	52.1	0	R701L (CGA→CTA)
spoT	0	0	0	9	R487H (CGT→CAT)
araB	0	0	8.2	0	Coding (559/1701 nt) Δ 1bp at 72,294
nadR	100	0	100	0	Coding (165/1233 nt) Δ1bp at 4,615,693
Membrane and Cell Wall:					
mrdA	0	0	0	43.5	D354A (GAC→GCC)
Transport:					
nmpC	0	0	0	47.1	Pseudogene (421/606 nt) T→G at 547,858
Others:					
ECB_01992	0	100	0	0	Coding (154/216 nt) +CAGC at 2,103,887
proQ	0	0	0	12.2	Coding (364-370/699 nt) Δ 7 bp at 1,893,742
thiC/rsd	56.5	0	0	0	Intergenic (-134/+100) A→C at 4,175,844
waaW	10.1	0	0	0	Coding (626-631/984 nt) Δ 6 bp at 3,738,225

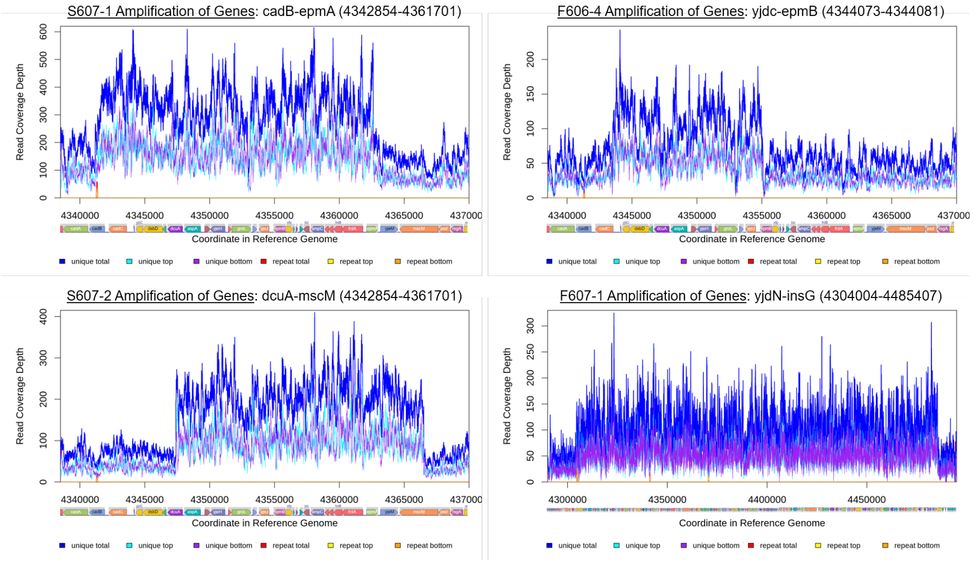

Figure S 6: Various amplifications of genes in 4,300,000 - 4,500,000 region for strains: S607-1, S607-2, F606-4, and F607-1. Strains S607-1, S607-2, and F606-4 have amplifications in smaller region between cadA and rsgA. While F607-1 has larger amplification encompassing this smaller region.

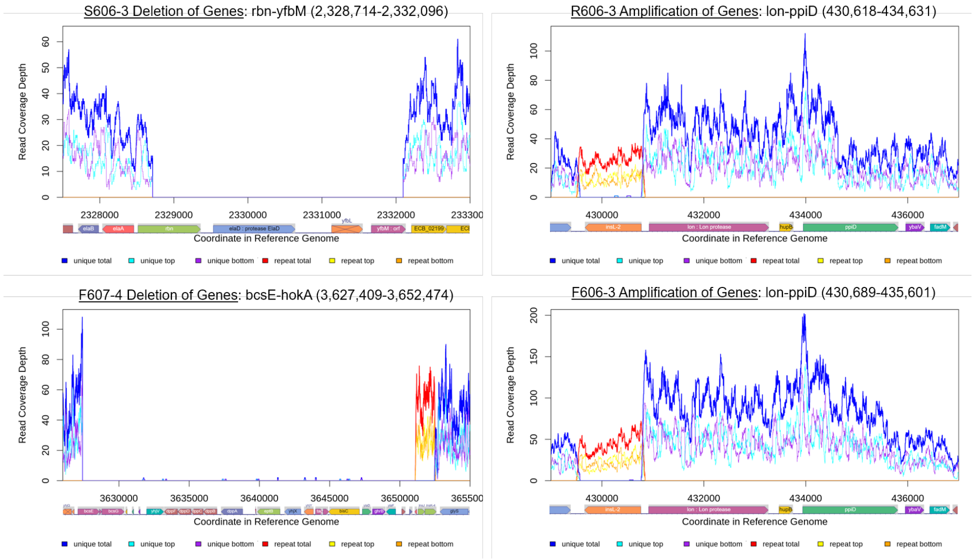

Figure S 7: Upper left: deletion of rbn-yfbM genes for S606-3 strain. Lower left: deletion of bcsE-hokA genes for F607-4 strain. Right: amplifications of lon-ppiD genes for R606-3 and F606-3 strains.

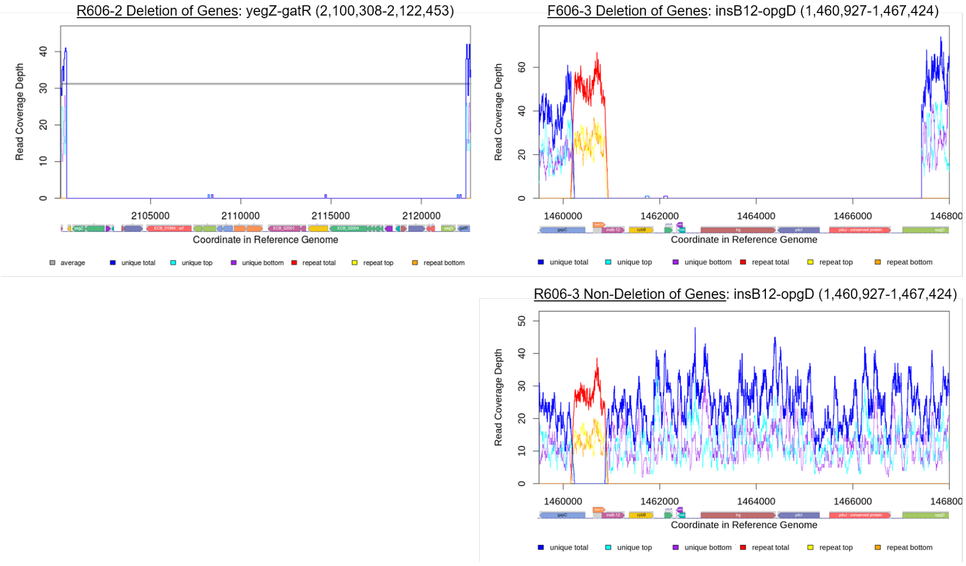

Figure S 8: Upper left: deletion of yegZ-gatR genes for R606-2 strain. Upper right: deletion of insB12-opgD genes for F606-3 strain. Lower right: lack of deletion of insB12-opgD genes for R606-3 strain.

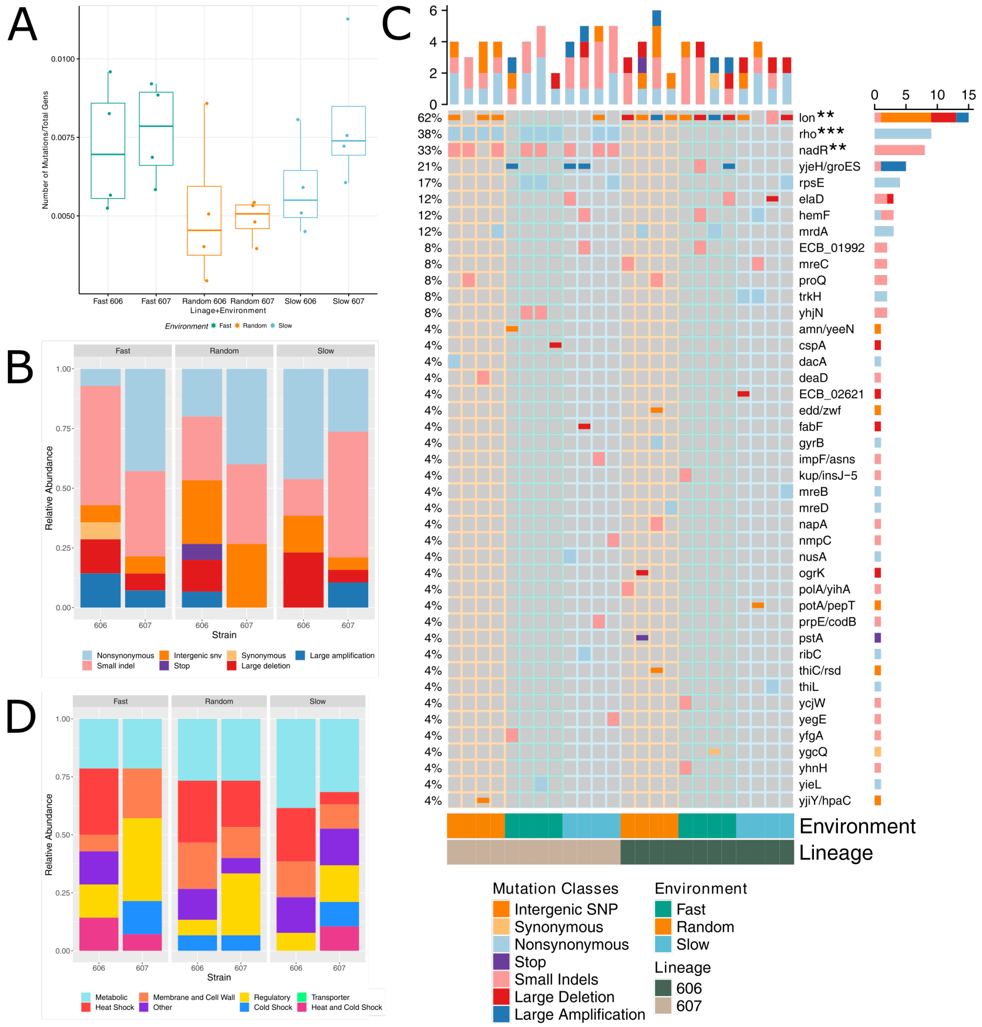

Figure S 9: Mutational composition of the clones of the final population evolved under fluctuating temperatures: A. Boxplot showing the comparison of the total number of mutations at the final time-point by strain and fluctuation regime. **B.** Relative frequency of each mutation by class at the final time-point by strain and fluctuation regime. 606 evolved clones have more large deletions and less non-synonymous mutations and small indels than the 607 clones (p-val = 0.021, 0.043, and 0.084, respectively). Random clones have more intergenic snps than the periodic clones (p-val = 0.013). Generalist clones have more small indels than the rest of the strains and specialists alone (p-val = 0.038 and 0.051, respectively), while specialists have more non-synonymous mutations than the rest of the strains (p-val = 0.068). **C.** Mutation incidence matrix of the clonal data. Mutations are coarse grained at the gene level. Large deletions and amplifications are counted once and collapsed to the repeatable gene. We use the Z-score to test significance of mutation enrichment between the evolved 606 vs 607 lineage clones for genes that had mutations in 3 or more clones, and *nadR* and *rho* mutations were significantly enriched for the 607 lineage clones (p-val = 0.013 with FDR = 0.04, and p-val = 0.00035 with FDR = 0.003, respectively). *lon* was enriched in periodic 606 clones (p-val = 0.011, FDR = 0.035). **D.** Relative frequency of each mutation by category at the final time-point by strain and fluctuation regime. 606 clones have less regulatory and cold shock mutations than 607 clones (p-val = 0.012 and 0.035, respectively). 606 clones also have more heat shock mutations than the 607 (p-val = 0.0082). Random clones have less mutations in genes that have both heat and cold shock functions than the periodic strains (p-val = 0.045). Clones classified as not significant also have less heat and cold genes mutated than generalists and specialists combined (p-val = 0.031), and generalists have more mutations these heat and cold shock genes than the rest of the strains (p-val = 0.052). Random clones have more heat shock mutations than the periodic clones (p-val = 0.024). Specialists have more metabolic genes mutated than the rest of the strains (p-val = 0.063). Strains classified as not significant have more heat shock mutations than generalists and specialists combined (p-val = 0.076).

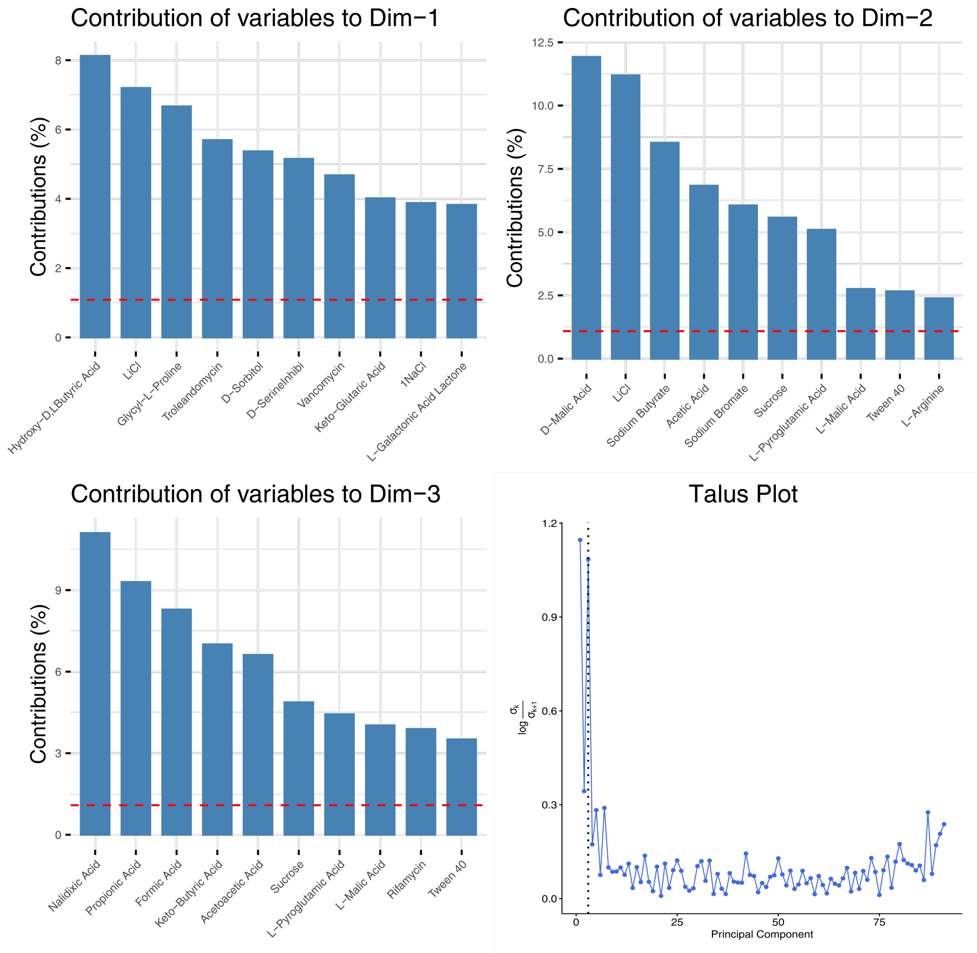

Figure S 10: Relevant principle components and their contributions: The contribution plots for PCA dimensions 1 - 3 show which wells are contributing the most to each dimension in the PCA. The talus plot shows random oscillations beyond three principal components, indicating that these are sufficient to explain the structure of the data. The dotted black line marks the number of components that have signal in the data.
